# Bird species richness and diversity responses to land use change in the Lake Victoria Basin, Kenya

**DOI:** 10.1038/s41598-024-52107-2

**Published:** 2024-01-19

**Authors:** Simon M. Mugatha, Joseph O. Ogutu, Hans-Peter Piepho, Joseph M. Maitima

**Affiliations:** 1 International Livestock Research Institute (ILRI), P.O. Box 30709, Nairobi, 00100 Kenya; 2 Ecodym Africa, P.O. Box 50901, Nairobi, 00200 Kenya; 3 Biostatistics Unit, Institute of Crop Science, University of Hohenheim, Fruwirthstrasse 23, 70599 Stuttgart, Germany

**Keywords:** Ecology, Zoology, Ecology

## Abstract

The increasing demand for cultivated lands driven by human population growth, escalating consumption and activities, combined with the vast area of uncultivated land, highlight the pressing need to better understand the biodiversity conservation implications of land use change in Sub-Saharan Africa. Land use change alters natural wildlife habitats with fundamental consequences for biodiversity. Consequently, species richness and diversity typically decline as land use changes from natural to disturbed. We assess how richness and diversity of avian species, grouped into feeding guilds, responded to land use changes, primarily expansion of settlements and cultivation at three sites in the Lake Victoria Basin in western Kenya, following tsetse control interventions. Each site consisted of a matched pair of spatially adjacent natural/semi-natural and settled/cultivated landscapes. Significant changes occurred in bird species richness and diversity in the disturbed relative to the natural landscape. Disturbed areas had fewer guilds and all guilds in disturbed areas also occurred in natural areas. Guilds had significantly more species in natural than in disturbed areas. The insectivore/granivore and insectivore/wax feeder guilds occurred only in natural areas. Whilst species diversity was far lower, a few species of estrildid finches were more common in the disturbed landscapes and were often observed on the scrubby edges of modified habitats. In contrast, the natural and less disturbed wooded areas had relatively fewer estrildid species and were completely devoid of several other species. In aggregate, land use changes significantly reduced bird species richness and diversity on the disturbed landscapes regardless of their breeding range size or foraging style (migratory or non-migratory) and posed greater risks to non-migratory species. Accordingly, land use planning should integrate conservation principles that preserve salient habitat qualities required by different bird species, such as adequate patch size and habitat connectivity, conserve viable bird populations and restore degraded habitats to alleviate adverse impacts of land use change on avian species richness and diversity.

## Introduction

The African continent is home to 20% of the earth’s bird species, totaling over 2000 species, with 90% being African endemics and the rest primarily winter visitors from the Palearctic region^1^. Sub-Saharan Africa is one of the locations on the earth with the greatest area of uncultivated arable land (*ca*. 2 million km^2^), accounting for about 50% of the global total^2^. One significant factor contributing to this uncultivated land is the presence of the tsetse fly (*Glossina *sp.); the primary vector for human sleeping sickness and animal trypanosomiasis^3^. This, plus the rapidly growing human population, the associated need for increasing food production and expanding socio-economic development, fuel a rising demand for tsetse eradication treatments, and land-use conversion in Sub-Saharan Africa. These changes are expected to significantly impact existing biodiversity. Therefore, it is imperative to understand the nature, magnitude and likely consequences of these changes. As of 2001, tsetse fly control operations covered about 128,000 km^2^ of Africa, representing 1.3% of the tsetse infested area on the continent^4^. These tsetse control efforts often involve clearing natural land cover in different parts of Sub-Saharan Africa^5^.

As tsetse control efforts expand to free up more arable land by reducing the incidence of trypanosomiasis, the use of chemicals and other tsetse control methods are having multiple adverse environmental impacts on biodiversity^6^. While non-chemical alternatives are advocated, they have yet to provide effective substitutes for insecticides. Even so, rising concerns about ecological damage and economic factors are driving a more discriminating use of insecticides in tsetse control efforts^7^. Not surprisingly, the impact of tsetse control on biodiversity has exhibited mixed outcomes. Certain control methods, such as bush clearing and organochlorine insecticide spraying, have led to the disappearance of many wild animal species, including of birds. Sheer clearing efforts have destroyed various plant species, hindered regrowth due to the use of arboricides and destroyed critical bird habitats. Overall, bush clearing, intensified land use, application of chemicals and increased cultivation and livestock populations drive the decline in wildlife populations, including of birds, with minor exceptions^7^.

However, natural landscapes are vital for birds. They typically host the highest bird species diversity, including rare, threatened, or unique species. However, human activities causing habitat attrition, fragmentation and degradation of natural habitats pose grave threats to bird conservation. Birds often struggle to adapt to these changes and are often unable to disperse to areas between habitat remnants or to adjust to altered habitat quality^8–10^. This makes it critical to address the broader impacts of habitat alteration, degradation and destruction driven by human population growth, land use changes, urbanization and industrialization^11^. Even so, avian conservation efforts are frequently concentrated on protecting threatened species, and conserving a few premier avian ecosystems (such as the Arabuko-Sokoke forest in Kenya). Clearly, it is equally important and critical to assess the conservation needs of birds facing mounting pressures from human population growth on land more generally, including in Sub-Saharan Africa.

Many birds fly across vast landscapes to access and exploit spatially distinct habitats. The distinctiveness and attractiveness of these habitats to bird species and other organisms is a function of their size and quality, largely mediated by biophysical factors, including land use change^12^. Changes in land use can and often substantially alter habitat extent and quality for birds. In aggregate, these modifications have fundamental adverse consequences for bird communities^13,14^. These modifications can result in habitats becoming too small to meet the minimum area requirements for certain species^15^, with adverse consequences for their survival prospects and population viability.

Perturbations caused by land use changes predispose birds to myriad risks, including localized population extirpations^16–18^. Intra- and interspecific variation in bird species’ responses and sensitivity to habitat alterations, range from local colonisations to local species extinctions^19,20^. Nonetheless, changes in bird species’ richness and diversity can provide valuable and reliable early warning signals of impending harmful habitat disruptions due to changing land use patterns^21^.

Rapid land use changes can adversely impact bird habitats and populations through the loss of native vegetation cover^22^. In particular, land use activities including settlements, agriculture and livestock production typically expand, leading to loss of native vegetation, after reductions in the prevalence of trypanosomiasis^23,24^. For example, in areas where tsetse fly control has been implemented, settlements, cultivation and livestock herding are the predominant drivers of land use and cover change in the Lake Victoria Basin in Kenya. Notably, within the lake basin human settlements have spread right up to the border fence of the Ruma National Park in Lambwe Valley. Moreover, between 2002 and 2004, over 27% of natural bushland was converted to cropland in Busia and Angurai, also within the Lake Victoria Basin^23^. The land use changes in Lambwe Valley, Busia and Angurai in western Kenya are concentrated in the most crucial biodiversity reservoirs. Such anthropogenic reduction in natural land cover often fundamentally alters richness and diversity of resident species^25^, often leading to the loss of high-quality habitats, altered food resources and an increase in low-value invasive herbs, suitable mainly for opportunistic and generalist fauna^26,27^. Thus, although sustainable land use should aim to balance developmental goals of enhancing human welfare with nature conservation^28,29^, there is little evidence to suggest this balance is being achieved in western Kenya.

This study was part of two larger projects focused on monitoring environmental changes resulting from tsetse fly control and the associated agricultural expansion. The two projects were implemented by the International Livestock Research Institute (ILRI) in collaboration with its partners from 1997 to 2004 in Kenya, Uganda, Ethiopia and Tanzania in East Africa. This study aimed to answer the question: how does land use and cover change in tsetse fly control areas affect Sub-Saharan African birds? To address this question, we compare and contrast bird species richness, diversity and composition between residual natural/semi-natural and disturbed (e.g., cultivated) areas targeted by the two tsetse fly control projects during 1997–2004 in the Busia, Angurai and Lambwe Valley sites, all located in the Lake Victoria Basin in western Kenya. More precisely, we compare human-dominated agricultural landscapes with adjacent natural/semi-natural landscapes as benchmarks to assess the nature and extent of changes in bird species richness, diversity and composition consequent upon land use changes^30,31^. This assessment is an important first step for in-depth and comprehensive assessments of the consequences of land use and cover change on avian species at larger spatial scales and longer temporal frames.

## Materials and methods

### Study sites

The study was carried out at three sites, one reference site, namely Lambwe Valley and two altered sites, namely Angurai and Busia. We classified each site into natural vs disturbed (human dominated, degraded, e.g., fallow and *Lantana *sp. dominated) landscapes. We characterize each site primarily in terms of location, vegetation types, topography, degree of fragmentation, patch sizes, rainfall amount (much lower in Lambwe) and spread and human-driven landscape changes. This is important to ensure that observed differences between the sites are not a consequence of pre-existing variation due to, say, climate or vegetation, rather than to human-driven landscape changes.

**Figure 1:**
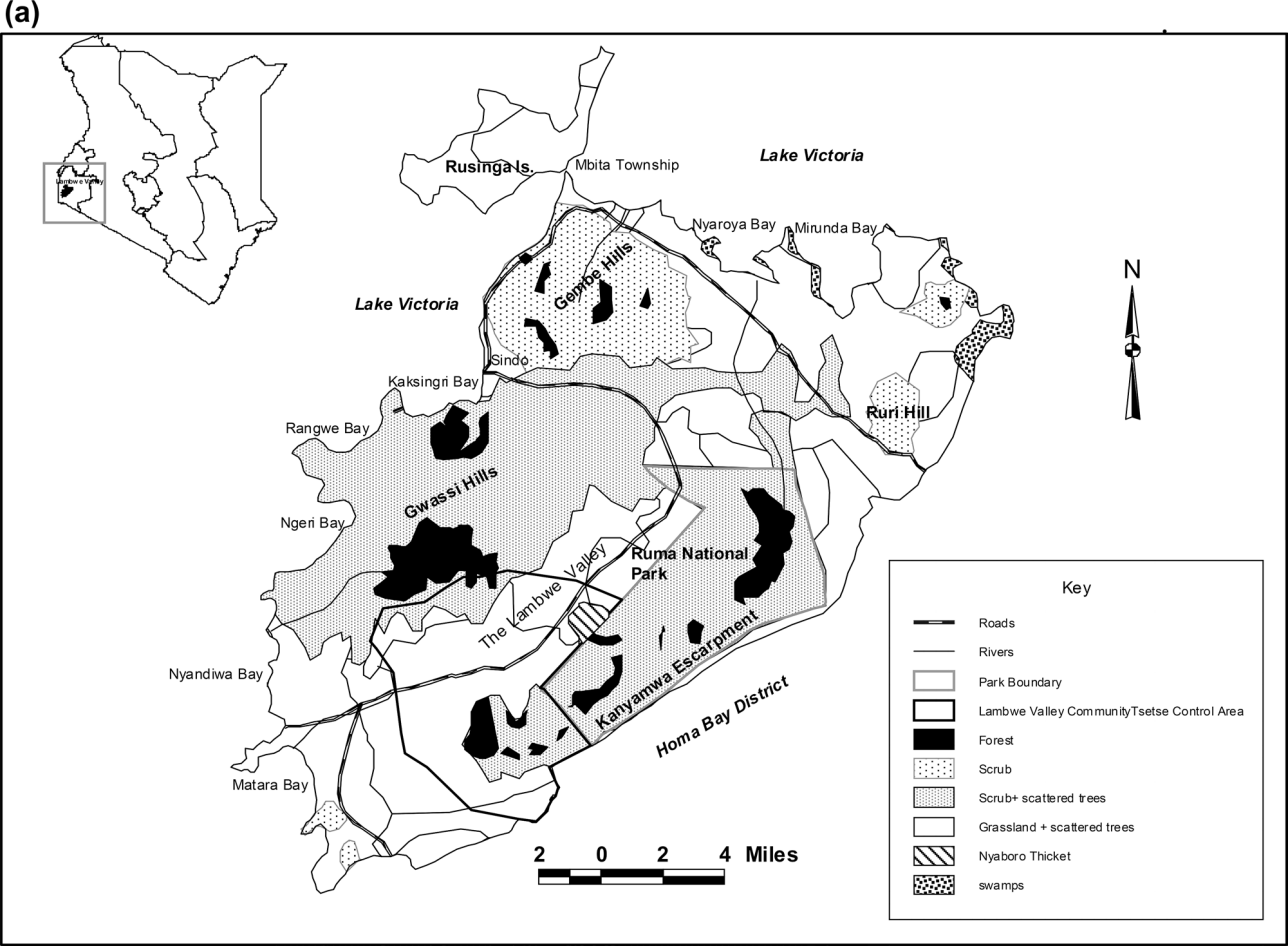

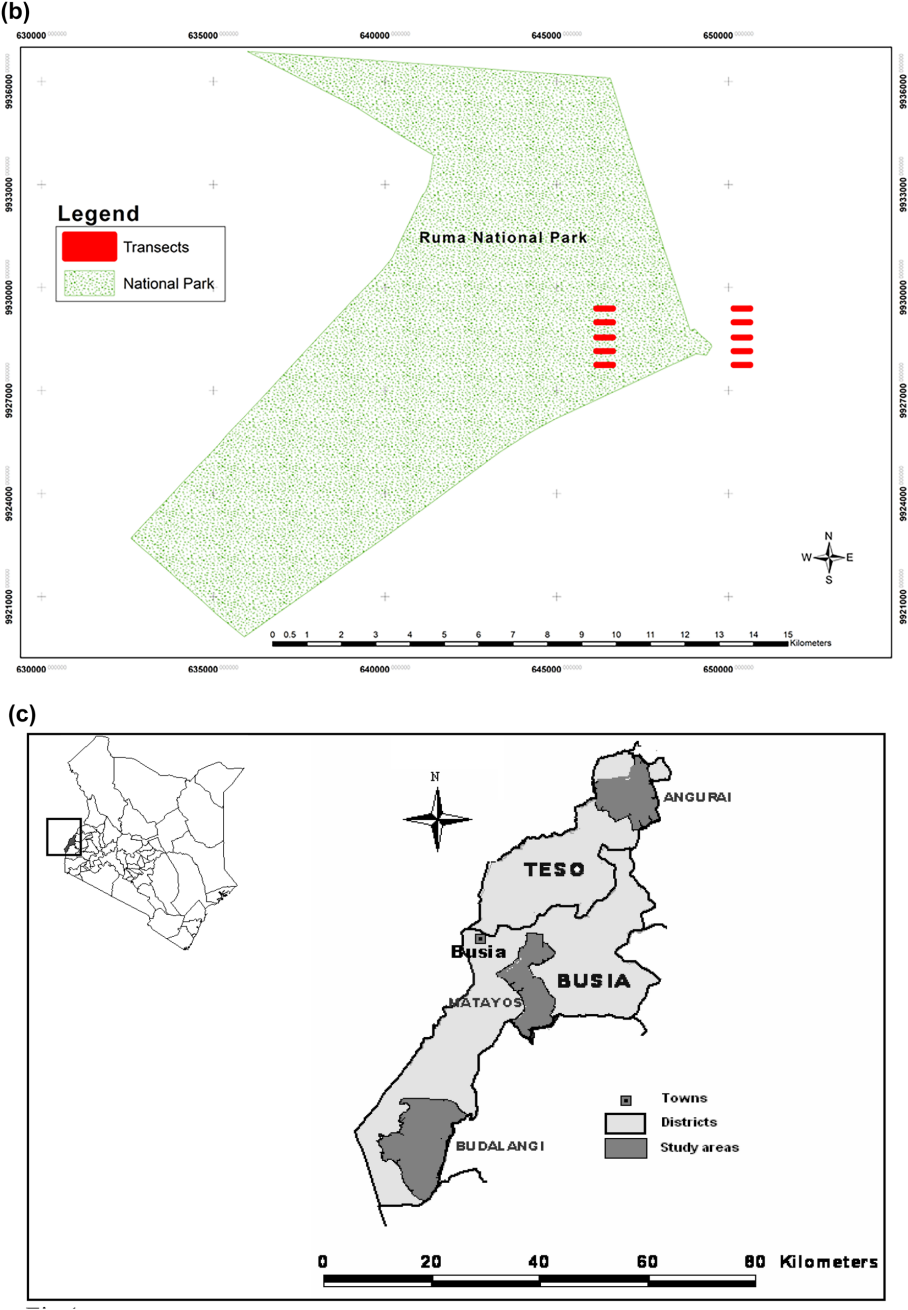
Map depicting the study sites located in western Kenya and featuring (**a**) Lambwe Valley and location of the Ruma National Park on the valley floor, (**b**) spatial locations of the 5 strip transects inside and 5 strip transects outside the Ruma National Park, and (c) Busia and Angurai.

Lambwe Valley, situated between latitudes 0° 30′ to 0° 45′S and longitudes 34° 10′ to 34° 20′E, is at an altitude of 1170–1750 m above sea level (a.s.l), and lies about 10 km east of Lake Victoria in western Kenya (Fig. 1a). Ruma National Park (120 km^2^), situated in the northern part of Lambwe Valley, is a nationally protected wildlife conservation area and home to the nationally rare roan antelope (*Hippotragus equinus langheldi*). The park was first established as the Lambwe Valley Game Reserve on 13 April 1966 through Gazette Notice No. 1308^32^ and later designated a National Park in 1983^33^. The park is located on the flat valley floor and is bounded by the Gwasi hills to the west, Kanyamwa escarpment to the southeast and Gembe and Ruri hills to the north. It is almost entirely enclosed by a wire fence erected in 1994 and surrounded by dense human settlements^34^. Lambwe Valley has a sub-humid to semi-arid climate with mean annual rainfall ranging from 1200 to 1600 mm. Rainfall is bimodal, with the long rains spanning March-June and the short rains covering September–November. The mean annual temperature ranges between 17 and 30 °C^35,36^.

In Lambwe Valley, the persistent threat of trypanosomiasis, transmitted by the tsetse fly *Glossina*, had historically limited human settlement. However, after extensive and prolonged efforts to control the tsetse fly population, the Lambwe Valley witnessed a notable upswing in its human population growth rate. The demographic shift was accompanied by rapid transformations in land use and vegetation cover and a remarkable three-fold expansion of cultivation in settled areas over a 50-year timeframe. This expansion resulted in a corresponding reduction in the extent of natural woody vegetation cover within the region^24^.

Consequently, land under cultivation in Lambwe Valley increased tenfold between 1977 and 1993, reducing the acreage of open grassland by 29% and of thickets by 41%^37^. The valley’s predominant vegetation communities are savanna grassland and woodland, with extensive acacia thickets and bushes. In Ruma NP the vegetation is relatively less disturbed except on the open grasslands, where seasonal or occasional burning is undertaken by park authorities to enhance grass palatability for wildlife^37^. The soils are largely ‘black cotton’ clay. The vegetation in the park is dominated by evergreen forest and wooded grassland consisting mainly of *Balanites aegyptica, Acacia drepanolobium and Acacia seyal* woodland or bushland. The common grass species in the park include *Themeda triandra* and *Setaria sphacelata* in the wooded grassland, *Themeda triandra* in the bushland and *Hyparrhenia filipendula* in the woodland^35^.

The park has a bird list that includes over 400 species and was listed as an Important Bird and Biodiversity Area (IBA) in 1999 (http://datazone.birdlife.org/site/factsheet/ruma-national-park-iba-kenya/details). It is the only protected area in Kenya in which the globally vulnerable and migratory blue swallow (*Hirundo atrocaerulea*) is monitored. *H. atrocaerulea* usually arrive in Kenya from their breeding grounds in southern Tanzania around April and depart in September and use the moist grassland for feeding and roosting. Some of the common birds found in the park are the crowned crane (*Balearica regulorum*), helmeted guinea fowls (*Numida meleagris*), marabou storks (*Leptoptilos crumenifer*), ibis (*Threskiornithinae spp.*), secretary bird (*Sagittarius serpentarius*) and quelea species (*Quelea *spp.)^38^. *Cisticola eximius*, a species once thought to be extinct in Kenya, was recently rediscovered in Ruma National Park.

Busia District, latitude 0° 26′ N and 34° 9′ E, is located in western Kenya at an altitude of 1160–1375 m a.s.l (Fig. 1b). The average annual rainfall ranges between 1270 and 1790 mm. By contrast, Angurai division, 0° 44′ N and 34° 19′ E, is located in the central and northern parts of Teso District in western Kenya at an altitude of 1300–1500 m a.s.l (Fig. 1b). The annual rainfall ranges between 750 and 1800 mm. The two rainy seasons in Busia and Angurai mainly occur between March and May and from August to October. About 50% of the annual rain falls between late March and late May, with an additional 25% during the short rains in August to October. The peak rainfall months are April and May. The annual mean maximum temperatures range from 26 to 30 °C whereas the annual mean minimum temperatures range from 14 to 22 °C^39^. The topography in both sites is undulating with hilly terrain. The landscape had very few trees dominated by fruit trees, including mango (*Mangifera indica*) and orange (*Citrus *spp.) trees and some wild tree species, including *Ficus* and *Makhamia*. Bushes were dominated by the shrub *Lantana camara* that grows in abandoned farmlands, roadsides, and bushes fringing streams and rivers^39^ and *Tithonia diversifolia*. *Lantana camara* is an invasive species. It is perhaps structurally more diverse than cultivated landscapes, but because it tends to cover any natural vegetation communities, it is often botanically equivalent to a monoculture. Therefore, *Lantana sp.* dominated areas are not considered natural but as either semi-natural or disturbed habitats. So, even where it is prevalent we do not consider it as a distinct land-use type because it is typically intermixed with many other plant species. Rough terrain, rocks and shallow soils characterize the sites, with short trees, shrubs, bushes and tall grasses representing most of the natural vegetation.

Agriculture is the main activity in both sites, with land parcels ranging in size from small (2 acres) to medium (20 acres), gradually increasing in size away from urban areas. Farmers cultivate cereals and cassava *Manihot esculenta* for their own consumption and the local market and sugarcane *Saccharum officinarum* and tobacco *Nicotiana tabacum* as the main cash crops. Farmers also keep both local cattle breeds and crossbreeds.

In Busia district the tsetse control initiatives had various impacts on land use, wildlife biodiversity and human livelihoods, resulting in a doubling of cultivated land between 1961 and 1997. Additionally, successful disease management increased the number of livestock zero grazing units, expanding the land covered by perennial grasses such as Napier grass *Pennisetum purpureum* and other fodder plants. The intensification of livestock production presents major challenges to nutrient recycling and soil fertility, biodiversity conservation and general ecosystem health^7^.

**Figure 2:**
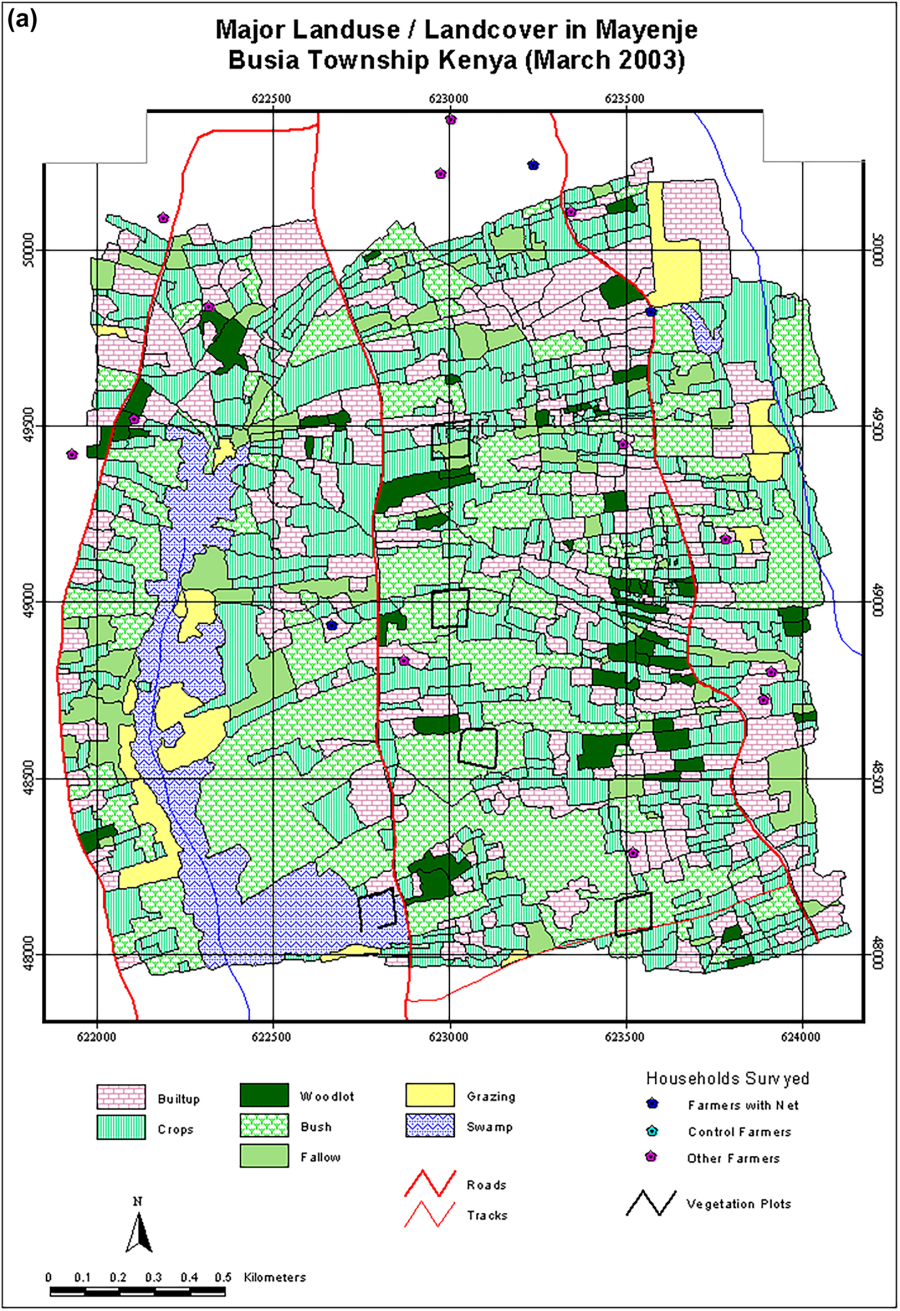

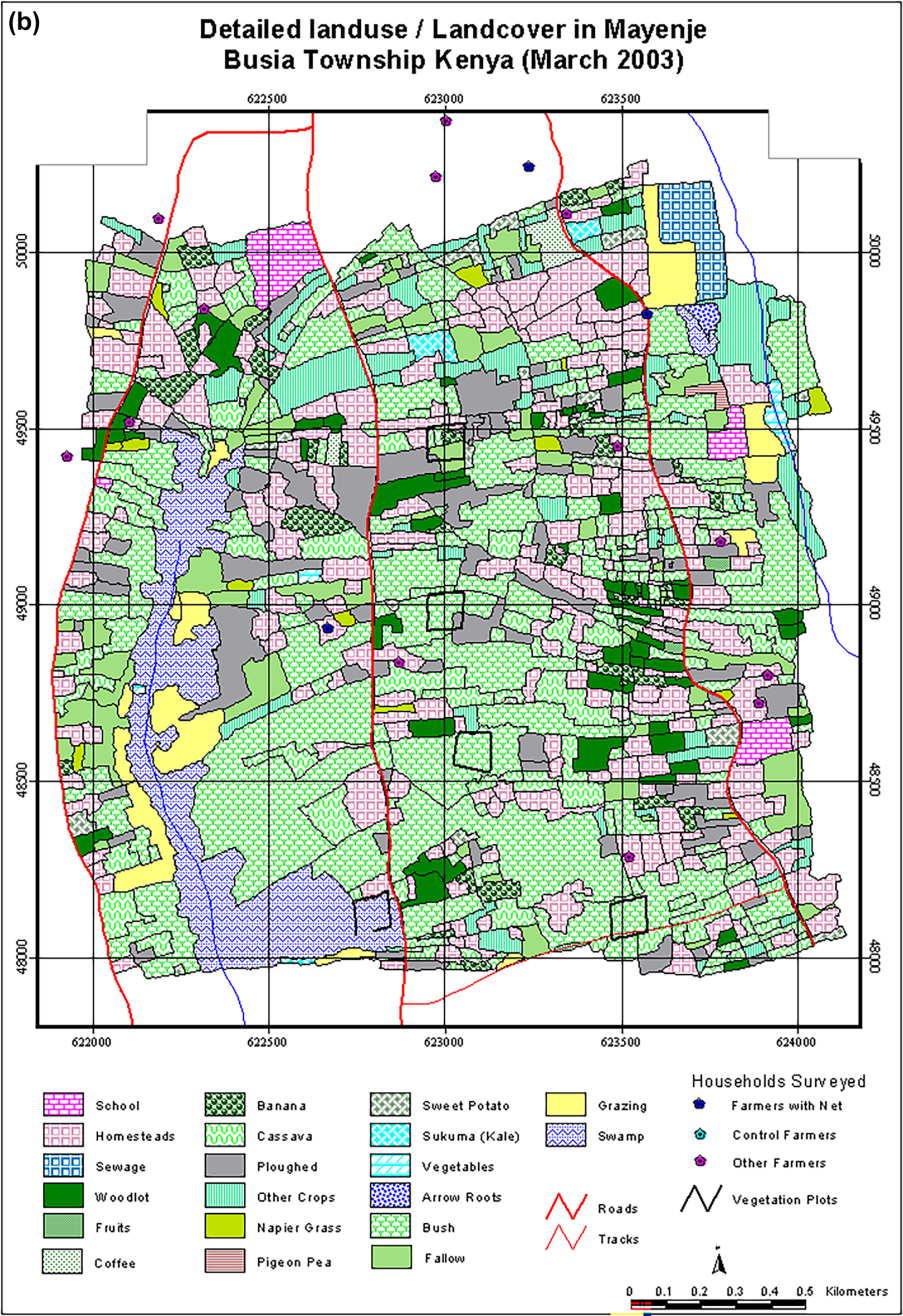
Map showing the (**a**) major or generalized land use classes and (**b**) detailed land use and land cover classes in Mayenje sub-location of Township location in Busia District, Kenya, in 2002. The study area is within 4 km of Busia town centre.

In Busia, natural vegetation covered 46% of the total area (4.3 km^2^). Although classified as disturbed land uses, and not regarded as natural vegetation, within the total area dominated by natural vegetation, there were patches of *Lantana camara* bushes (26.3%) interspersed with fallows (6.1%). Moreover, grazing lands (3.3%), swamps and related vegetation (6%) and woodlots (4%) occurred within the natural vegetation. Swampy areas were used intensively for grazing and as sources of thatch grass and firewood. In contrast, the modified landscape occupied 53%, with cultivation covering 30% and settlements and other infrastructure occupying 23%. Of the cultivated area, cassava occupied about 10%, a variety of annual crops 15% and perennial crops 5%. Perennial crops, including arrowroots *Maranta arundinacea*, sugarcane and vegetables were grown within homesteads and parts of the swamps, making the actual cropland area exceed 30%. We also identified and mapped small pockets of Napier grass used as animal feed in zero-grazing enterprises (Fig. 2a and b).

**Figure 3:**
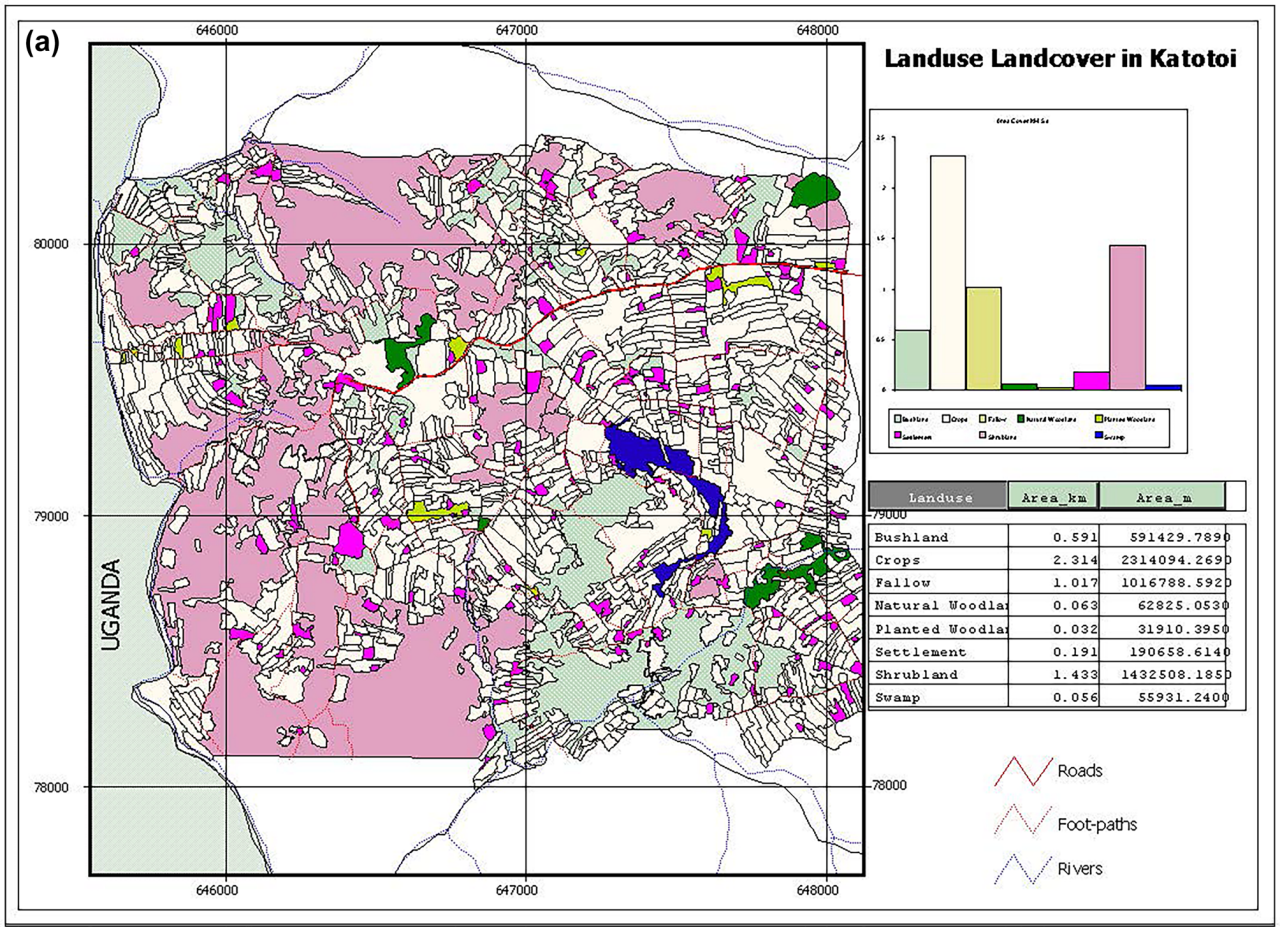

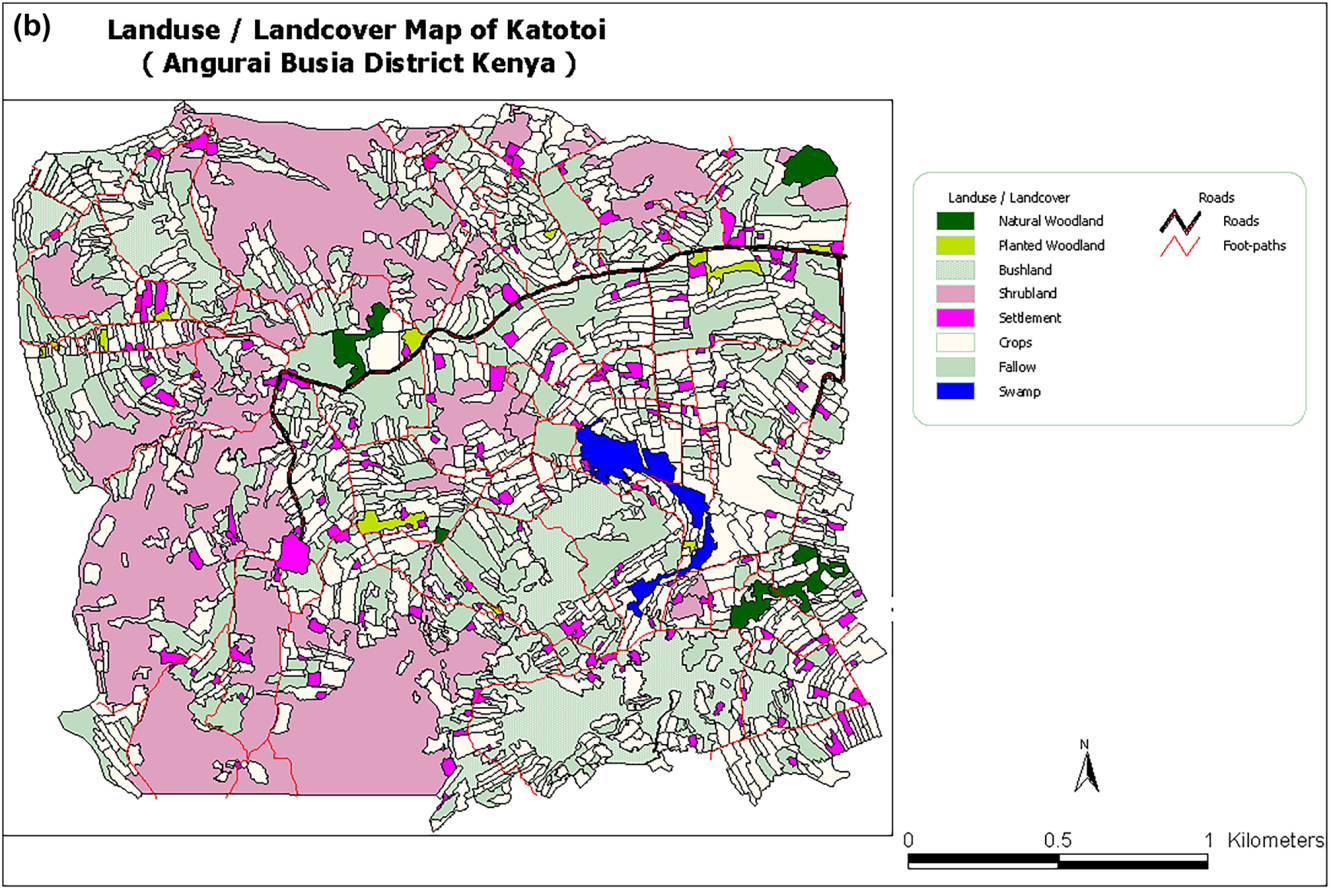
Map showing (**a**) land use and land cover classes, areal coverage of each class and geographical coordinates and (**b**) zoomed in land use and cover classes in Katotoi sub-location in Angurai Division of Teso District, Kenya, in 2002.

In Angurai, the dominant natural vegetation consists of short trees, shrubs, bushes and tall grasses. The tree species were exemplified by *Albizia coriaria*, *Combretum machowiancum*, *C*. *molle* and *Ficus kitubalu*, shrubs by *Acacia hockii* and *Heeria reticulata* whereas grasses by *Cymbopogon excavates* and *Halopyrum mucronatum*. Out of the total area (5.76 km^2^), land use was distributed as follows: crops occupied 41.0%, shrubland (25%), fallow (18%), bushland (10%) and settlement (3%). Planted and natural forests and swamps jointly occupied 3%. Angurai, like other areas in western Kenya, underwent rapid land use changes. Between 2002 and 2004, shrub land reduced by 27%, representing a cumulative loss of 1.48 km^2^ to cultivation and livestock grazing, the largest change. Land conversion from natural vegetation to crop cultivation accounted for over 80% of the total land use change. This transformation involved the expansion of crops such as a mixture of finger millet *Eleusine coracana* and sorghum *Sorghum bicolor* (25%) and maize *Zea mays* (13%). The area dedicated to cassava, tobacco and banana *Musa* spp. decreased, but that occupied by groundnuts *Arachis hypogaea* changed little (Fig. 3a and b). About 16% of Angurai experienced other land use changes, which we do not consider further in this study.

Busia and Angurai have varying terrains, ranging from steep, to low/ flat areas depending on the gradient. Most cultivation occurs in moderately steep to low/flat areas, such as hilltops and lowlands near swamps, rivers and streams. Steep slopes are mostly covered by natural vegetation, with minimal crop cultivation, mainly sorghum and maize. Extensive harvesting of wood from natural vegetation leaves behind only short, low-value trees. The high demand for fuel wood is driven primarily by the need to dry construction bricks, tobacco leaves and by domestic use.

### Land use and cover mapping and assessing land use and cover change

Hand held Global Positioning System (GPS) devices and topographic maps were used to map the three sites and distribution of various land use patterns and cover types at the farm level, providing baseline indicators of land use and cover. Information on land use was also obtained from district topographic maps. GPS tracks were taken around each land use and cover types (crops, settlement and fallow areas), marking them as waypoints. In Busia, for instance, a team of five individuals conducted land use and cover mapping over a 10-day period from 4 to 15 March 2003. They covered about 4.3 km^2^, by walking a total of 266 km to delineate boundaries of 835 polygons representing various land use and cover types, including cultivated areas, built-up areas, and natural areas.

Subsequently, the GPS data were downloaded to a computer for further processing and mapping. The untracked areas represented non-cultivated zones, where vegetation was characterized and described. GPS readings were also taken for the plots where samples had been collected. The coverage of each land use and land cover type was estimated and described from the maps. Comprehensive land use and land cover maps of the study area were generated, providing detailed information about cultivated and non-cultivated areas, crop types, natural vegetation, fallow lands, water resources, settlements, public buildings, roads, and more. The mapped land use patterns were overlaid onto topographic maps and supplemented with remote sensing imagery to detect and assess changes in land use and cover over time. Each site was categorized into two main types: natural/semi natural vegetation (natural) and modified/settled/cultivated (disturbed) land uses. Furthermore, areas mapped for land use at all the sites were also surveyed for bird species using binoculars (Leitz 10 × 40 122 m/1000 m range).

### Bird sample surveys

In the Lambwe Valley site, the presence or absence of bird species was established using 10 walking transects. These transects were each 500 m long and 20 m wide. This survey was conducted from 17 to 20 August 1997^40^. Out of the 10 transects, five were positioned within the Ruma National Park and the other five were situated in human settlement areas. Within each of the two land use types, two transects were placed in natural thickets and two in open grasslands. The transect locations were carefully selected to accurately represent the natural and semi-natural (grasslands, thickets and grassland-thicket in the National Park) or disturbed (grassland, thickets and grassland-thickets in settled areas) habitat types in each land use type, and minimize potential biases associated with transect selection. The sampled habitats thus reflected the available habitats along each transect within each land use type. The natural transects were entirely confined within the Ruma National Park.

Along each transect, birds were counted twice in the morning and twice in the afternoon on different days. Each transect was surveyed using timed walks along its centerline. All the birds on the transect were recorded from the transect centreline to minimize potential biases and confounding effect of detection probabilities. The timed transect walks spanned three hours in the morning, between 6:30 a.m. and 9:30 a.m., and two hours in the evening, between 3:30 p.m. and 6:30 p.m. During these walks observed bird species were recorded. Concerted effort was made to search for and detect cryptic bird species during the surveys, including aerial ones (swift and swallows) and birds seen flying above. This survey procedure was repeated consistently over two consecutive days for each transect.

Birds were detected, identified and counted using both visual observation and voice recognition. On each occasion, the species observed on a particular transect and land use were recorded and aggregated to indicate frequency. Birds sighted outside the designated transect area were counted and recorded separately whereas those sighted while flying over the transect were counted as birds flying over the transect. To mitigate potential observer biases, all the surveys were carried out by the same observer and recorder. For standardization, the International Ornithological Committee (IOC) world checklist^41^ was used as a reference for both the common and scientific names of the detected bird species.

In Angurai and Busia study sites, birds were surveyed on eight plots (50 m × 100 m) per site. Except for one plot (WDBS, Table  S1) which had neighbouring native cover, all the other plots, were either cultivated or partly cultivated. Even plots that contained native species were so greatly altered and surrounded by cultivation and were too small to support viable bird biodiversity on their own. The Angurai site had changed dramatically over the preceding recent decades, with virtually all available land converted to cultivation and natural cover removed. As a result, the remaining vegetation cover was insufficient to support most of the woodland savanna bird species that had once inhabited the area. Similarly, the Busia site had undergone extreme land cover changes, such that almost all the immediate vicinity of the plots had been cultivated. The only major exception was a huge swamp located adjacent to Plot 7 (Table S1). This swamp remained unaltered, and its avifauna had probably not changed much over time^42^.

At the time of the survey conducted in Angurai from 10 to 12 August 2004, and in Busia from 14 to 15 August 2004, no migratory birds were present in the area, as they were still in their Eurasian breeding grounds. Typically, many birds, predominantly insectivores or carnivores, but not obligate frugivores or granivores, pass through the area from October to December. While a few birds choose to stay, most pass through the area again in March to early May. As a result, all the recorded bird species in the area during the survey were residents of the immediate vicinity, if not found directly on the surveyed plots themselves. The only exception to this pattern was the barn swallow *Hirundo rustica*, which is an early arrival in the region, typically appearing from early July onwards^42^.

The GPS coordinates for all the surveyed plots and detailed descriptions of vegetation on the plots at Busia and Angurai are provided in Table S1. The plots in the Angurai and Busia sites were visited over a period of seven days and the bird species sighted on each plot during each visit recorded. Most of the survey was carried out from one spot in each plot at the optimal observation times of early morning and late afternoon. In the warmer parts of the day when birds were inactive and silent, the plots were systematically patrolled to search for, locate and record the birds. Each of the natural plots was visited early in the morning or late afternoon. Conversely, the disturbed plots were surveyed both in the morning and during the warmer parts of the day. Most of the species observed on a particular plot were recorded during the morning and afternoon visits^42^.

Detected species were aggregated according to their feeding habits following^43^. To assess the relative species richness of each land use type, the total number of species observed within a particular land use type was compared to the overall number recorded for the entire site during the survey. All the survey data were aggregated at the transect or land use level for analysis. The presence/absence data for all the sighted species are summarized at the plot or transect level in S1 and S2 Datas and at the site and land use levels in S3 Data.

### Assessing influences of range size, foraging style and threat level on species use of disturbed and natural areas

To evaluate the impact of range size, foraging style and threat level or trend on bird species' use of disturbed and natural areas, we gathered data on these variables from the BirdLife Datazone website (https://datazone.birdlife.org/species/search). Specifically, we collected details on each species' approximate breeding range size, foraging style (non-migratory or migratory—which includes nomadism, partial migration, or altitudinal migration)—and threat level or trend, classified as decreasing, stable, or increasing (S4 Data). Using this information, we computed the average range size and its standard deviation for all the sighted bird species. Additionally, we summed up the total number of bird species sighted in the disturbed and natural habitats. These calculations were performed for each unique combination of study site, foraging style and threat level or trend. This approach allowed us to analyze how these factors influence avian species' preferences for the disturbed versus the natural land use.

### Addressing potential limitations in the sampling survey methods and sample sizes

Several sources of variation, error, or bias could potentially impact our results. While we made significant efforts to address them during field sampling and statistical analysis, certain sources of potential bias such as habitat heterogeneity, or bird behaviour, cannot be fully mitigated by these methods. Here, we highlight these potential sources of variation, error, or bias and describe our efforts to minimize them. We keep these factors in mind when interpreting the results.

Our sampling method and data have potential implications for the results and their interpretation. Therefore, when discussing the results, we also note instances where they may not be representative of the wider landscapes. The strip transect and plot surveys we used do not fully account for potential variations in detectability across different sites or habitat types. However, it is imperative for avian surveys to minimize detection errors and strive for representativeness. It is well known that, bird counts can be significantly impacted by detectability biases, such as the possibility of missing birds due to limited visibility caused by factors such as dense vegetation or rugged terrain. Detection errors are closely related to the number of surveys conducted, the extent of the area covered and the number and variety of species recorded. While the relatively long transects, and repetition of the surveys on the second day, likely minimized some potential detection errors, four surveys conducted over 48 h along the same transect such as in Lambwe Valley may still be insufficient to detect species that only occasionally use each site. This limitation is particularly relevant for wide ranging species with extensive home ranges such as raptors. It is well known that as the number of surveys increases, and the coverage expands, the number of recorded species tends to increase until observers eventually record nearly all the species present. This suggests that our sampling may potentially have underrepresented certain categories, such as the natural category in Lambwe Valley. For, if more extensive surveys were done in Lambwe Valley, there would likely be many more species detected given that the area is known to host around 400 species.

Moreover, the challenge is particularly pronounced for species with extensive home ranges such as raptors, where the probability of detection in any single survey is quite low, and many surveys and much longer transects would probably be required to capture all the species in an area. Efforts to minimize detectability bias often involve the adoption of specialized sampling techniques like distance sampling. However, these methods typically require sighting a minimum of 60–80 distinct groups to achieve accurate estimates of population size and related parameters^44^. This can be hard to achieve in practice for rare species, which often are the ones of great conservation concern. Moreover, even distance sampling is unlikely to be effective for cryptic and elusive, rare or wide-ranging species that are hard or may be unavailable to detect. Detection probabilities for birds can also vary over the breeding season and between seasons due to movements. There can also be sporadic, large concentrations of birds, especially migratory species, in specific localities such as agricultural areas during the non-breeding season. For example, some species such as raptors can exhibit considerable fluctuations in population density depending on the time of year. In many areas, they are easier to detect at mid-day, highlighting that detectability can also vary both seasonally and diurnally.

Given detection probability varies by habitat type, differences between surveys may partly reflect differences in the types of habitat surveyed. If sampling effort does not adequately cover the full spectrum of available habitats, then some species will simply be missed because the whole suit of available habitats, such as vegetation types and topographic features, will not have been adequately sampled. This is particularly pertinent where the array of habitats is diverse, encompassing various botanical and topographic features, whereas in relatively uniform agricultural regions, this issue may present fewer challenges. These considerations can also impact the likelihood of detecting similar proportions of the species present in different areas. Furthermore, the situation can be further confounded if the sampling strategy does not proportionally represent the diverse habitats available within different land-use categories.

Our sample sizes were admittedly rather small given that biological communities are often inherently substantially variable, making large sample sizes undoubtedly desirable. However, our level of sampling intensity should suffice for detecting differences among common guilds or individual species, and at the very least, for overall richness during the survey period. For finer resolutions, such as for rare guilds or individual species, where formal comparisons may be tenuous at best, and differences between guilds, areas, or between natural and disturbed habitats may simply reflect chance encounters or sampling effects, we present the counts only in the supplemental materials. This is in contrast to large drops in species richness between the disturbed and natural land use categories such as those recorded for Busia and Angurai. Where we report small differences, for example, in the number of species within guilds that are only represented by a few species in each area, we provide statistical evidence to establish their significance based on our sampling method. This is crucial because differences in the number of species, such as one-species difference, between guilds in different areas, say, could easily be a result solely of sampling variability. As another example, whereas the differences in insectivores and granivores are represented well enough, that for raptors may require strong supporting evidence.

While we statistically compare various bird characteristics across sites, this is challenging due to inherent dissimilarities at landscape scales. Achieving identical conditions for a rigorous statistical comparison, except for the specific variable under investigation, is virtually unattainable in natural field conditions at landscape scales.

### Statistical data analysis

The data were processed and the richness and diversity of various bird species in the two land use types in the three sites analyzed using multiple methods. Bird species diversity was calculated using the Shannon–Wiener index (H′)^45^ and the indices tested for significant differences between sites and land use types^46^. The similarity of bird communities between land use types was tested using the Sørensen index (Ss)^47^.

A quasi-Poisson regression was used to test for differences in the expected proportion of bird species in each feeding guild between land uses, sites and site-by-land use interaction. The generalized linear model assumed the number of bird species in each feeding guild and land use followed a quasi-Poisson distribution and used a log link function to relate the expected species richness to the two main effects and their interaction. The logarithm of the total number of species tallied in each site was used as an offset in the quasi-Poisson regression model to derive numerical proportions of the total number of species in each feeding guild and land use relative to the total number for the corresponding site. The quasi-Poisson model explicitly allowed for overdispersion to account for potential flocking of birds. We used raw, Pearson and studentized residuals and outlier diagnostics to assess the goodness-of-fit of the model. For each type of residual, we plotted the residual against the linear predictor, quantile–quantile plots, box plots and a histogram with the normal density overlaid. These suggested a reasonable model fit except for six observations of species richness in the natural land use that appeared larger than expected. Since the interaction between land use type and site was significant, we partitioned (decomposed) it into its simple effect slices^48^ to compare the expected proportion of species between the two land uses for each site and the three sites for each land use. The model was fitted using the restricted maximum quasi-likelihood method in the SAS Glimmix Procedure^49^.

## Results

### Species richness

**Figure 4:**
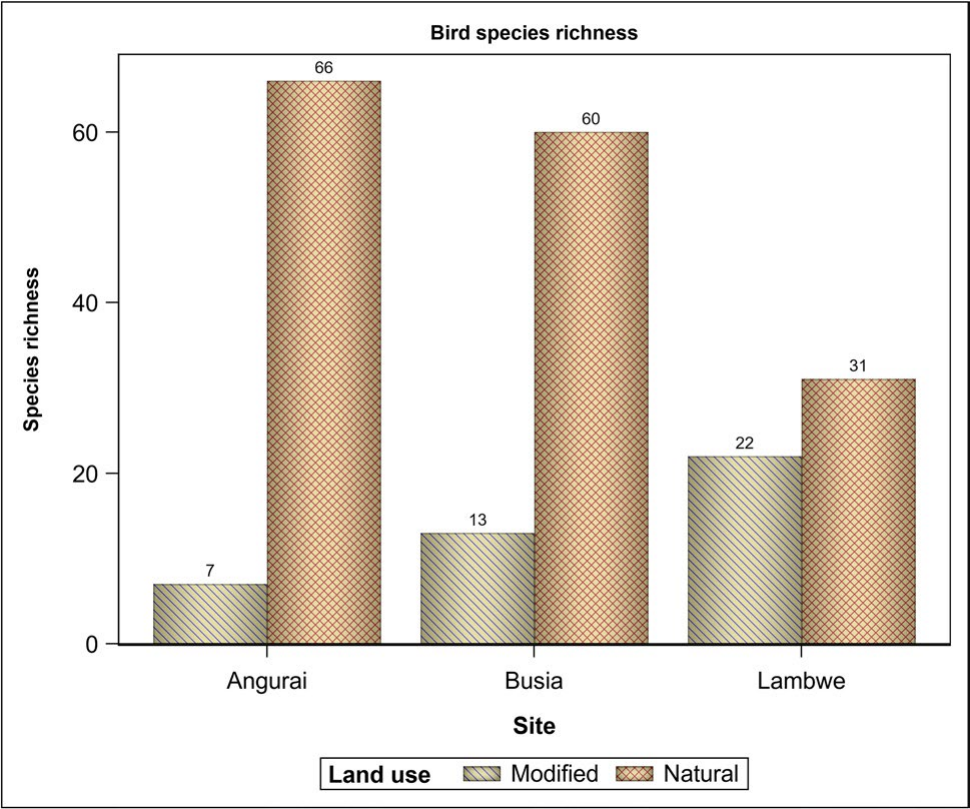
Bird species richness in Angurai (10–12 August 2004), Busia (14–15 August 2004) and Lambwe Valley (17–20 August 1997) sites.

A total of 168 different bird species were detected in Busia (*n* = 68 species), Angurai (*n* = 61) and Lambwe Valley (*n* = 53) (Fig. 4). For the natural land use, species richness was similar between Angurai and Busia but significantly higher (*P* < 0.05) for both sites than for Lambwe Valley. For the disturbed land use, species richness was comparable across all the three sites. Species richness was higher in the natural than the disturbed land use across all the three sites and was the highest for Angurai, middling for Busia and the lowest for Lambwe Valley. In contrast to the natural land use, bird species richness in the disturbed land use was the greatest for Lambwe Valley, intermediate for Busia and the lowest for Angurai. Of the three sites, Busia had moderate species richness both in the natural and disturbed land uses.

### Species composition

Some bird species occurred in both the natural and disturbed land uses in Busia (92%) and Angurai (71%). But others occurred only within the confines of the disturbed land use in Busia, namely *Poicephalus cryptoxanthus* and in Angurai *Halcyon chelicuti* and *Serinus sulphuratus*. However, species sighted in the natural land use were completely different from those sighted in the disturbed land use in Lambwe Valley (Table S2). Although the majority of birds occurring in the disturbed land use also occupied the natural land use, only a small proportion of birds inhabiting the natural land use occurred in the disturbed land use in Busia (20%) or Angurai (7.6%).

### Species diversity

**Figure 5:**
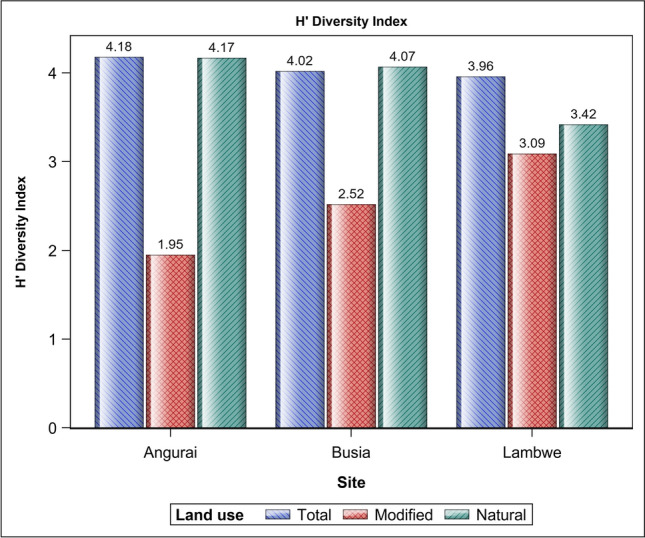
Shannon–Wiener Diversity Index (H′) of bird species in natural and modified landscapes in Angurai (10–12 August 2004), Busia (14–15 August 2004) and Lambwe valley (17–20 August 1997) sites.

The Shannon Wiener diversity index (H′) showed bird species diversity to be consistently higher in the natural than the disturbed land use across all the three sites (Fig. 5). For the natural land use species diversity was similar between Angurai and Busia but significantly higher (*P* < 0.05) for both sites than for Lambwe Valley. For the disturbed land use species diversity was comparable across all the three sites. The natural land use in Angurai had the highest species diversity relative to the disturbed land use across all the sites. Although Angurai had the highest recorded bird species diversity and richness in the natural land use, it also had the lowest species diversity and richness in the disturbed land use across all the sites. Similarly, the bird species diversity in the natural land use in Busia was greater than the intra-site diversity, or the diversity on the disturbed land use in that area. On the other hand, Lambwe Valley had the smallest difference in species diversity and richness between the natural and the disturbed land uses among all the three sites. The intra-site differences in species diversity between the natural and disturbed land uses in Angurai, Busia and Lambwe Valley were all statistically significant.

### Species guilds, richness within guilds and similarity index

All the recorded bird species belonged to 11 feeding guilds that were unevenly distributed between the two land uses across the three sites (Table 1). A total of 11 guilds were observed in the natural land use and nine guilds in the disturbed land use. The natural land use had 10 guilds in Angurai and eight each in Busia and Lambwe Valley whereas the disturbed land use had four guilds in Angurai, six in Busia and eight in Lambwe Valley. Insectivores and granivores were the two most diverse and abundant guilds in all the sites and land uses. In contrast, the insectivore/wax feeder, comprising *Indicator minor*, and the nectarivore, comprising *Cinnyris pulchella,* were among the least widely distributed guilds across the three sites. The insectivore/wax feeder guild was confined to the natural land use in Busia and Angurai, whereas the nectarivore guild was observed in both the natural and disturbed sites in Lambwe Valley.

**Table 1:** Bird species richness in each feeding guild and the Sorensen’s Similarity Index (Ss) of birds in natural and disturbed land uses in Angurai (10–12 August 2004), Busia (14–15 August 2004) and Lambwe Valley (17–20 August 1997) sites.

Species guild	Angurai				Busia				Lambwe Valley				Grand total
	Disturbed	Natural	Total	Ss	Disturbed	Natural	Total	Ss	Disturbed	Natural	Total	Ss	
Carnivore	0	3	3	0	1	1	2	–	3	2	5	0	10
Frugivore	1	4	5	0.44	2	3	5	0.33	1	4	5	0	15
Granivore	3	20	23	0.22	4	18	22	0.38	3	2	5	0	50
Insectivore	2	30	32	0.15	4	27	31	0.4	10	19	29	0	92
Insectivore/carnivore	0	1	1	–	0	0	0		1	0	1		2
Insectivore/frugivore	0	1	1	–	0	0	0		1	1	2		3
Insectivore/granivore	0	3	3	0	0	3	3		0	0	0		6
Insectivore/nectarivore	1	7	8	0.29	3	4	7	0.57	0	1	1	0	16
Insectivore/wax feeder	0	1	1	0	0	1	1	0	0	0	0		2
Nectarivore	0	0	0	–	0	0	0		1	1	2	0	2
Omnivore	0	1	1	0.25	1	7	8	0.18	2	2	4		13
Grand total	7	71	78		15	64	79		22	32	54		211

**Figure 6:**
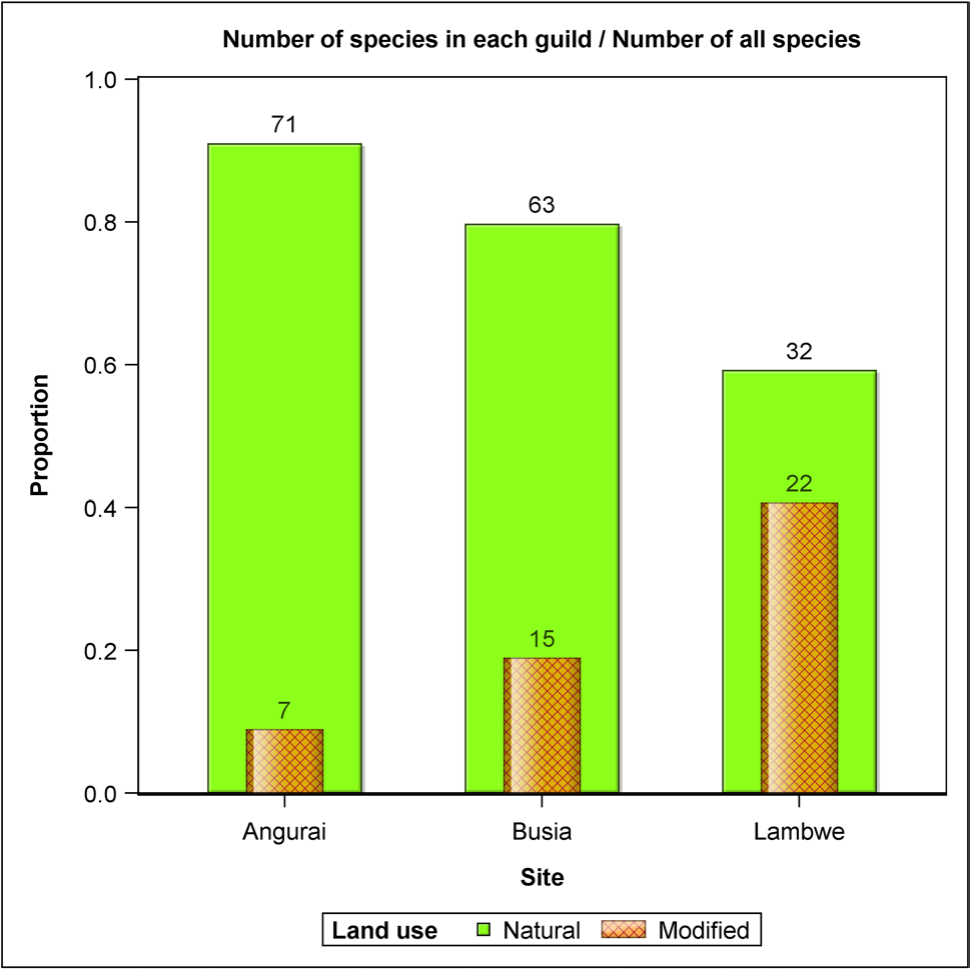
The proportional distribution of bird species richness between land use types at Angurai (10–12 August 2004), Busia (14–15 August 2004) and Lambwe Valley (17–20 August 1997) sites.

**Figure 7:**
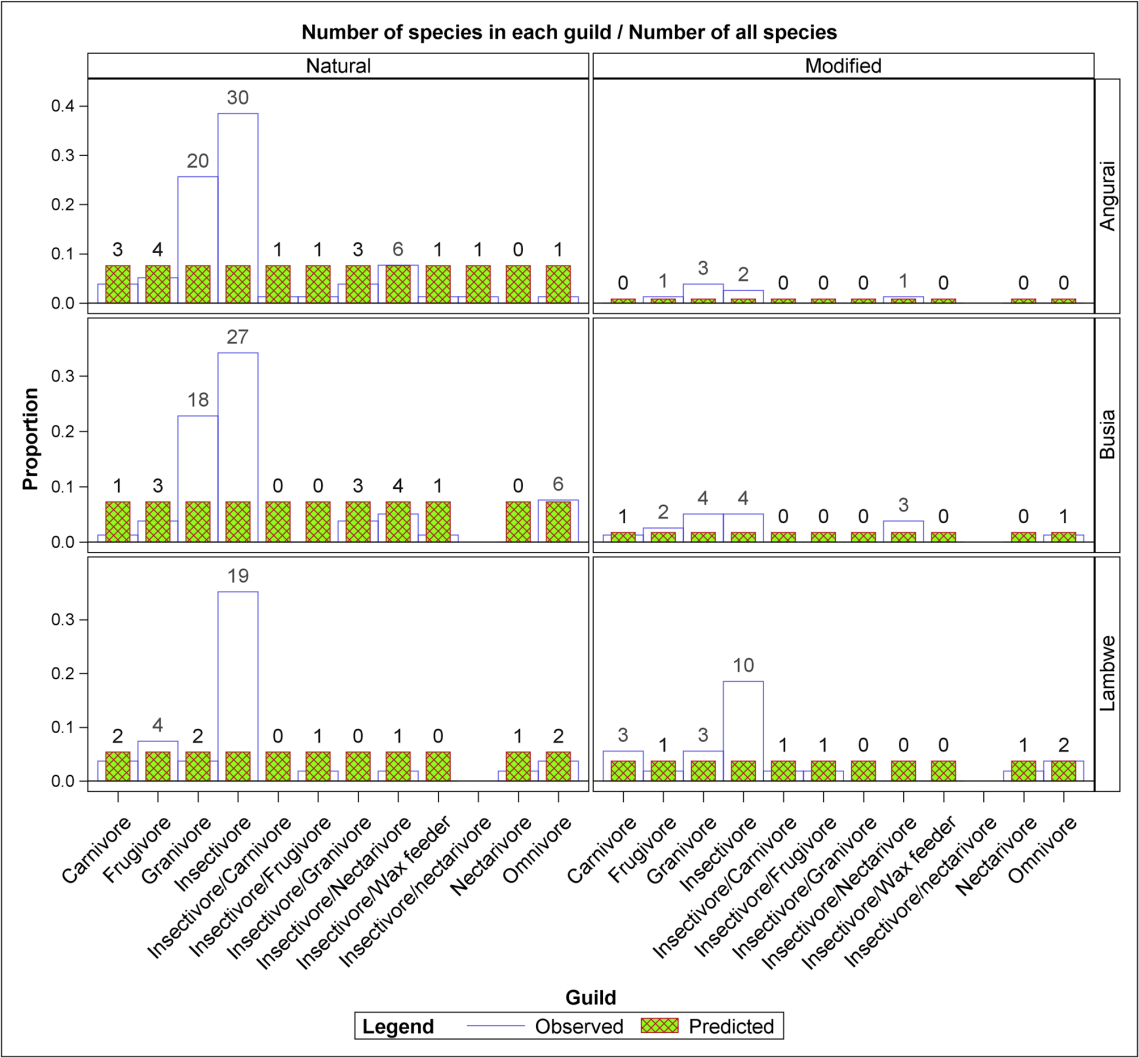
The proportional distribution of the observed and expected bird species richness grouped by feeding guild between land use types at Angurai (10–12 August 2004), Busia (14–15 August 2004) and Lambwe Valley (17 August 1997) sites.

All the nine guilds sighted in the disturbed areas also occurred in the natural land use. Conversely, two guilds, the insectivore/granivore and the insectivore/wax feeders occurred only in the natural land use (Table S2). Guilds occurring in the natural land use had significantly higher species richness than those occurring in the disturbed land use. There were significantly more species in the natural than the modified landscapes in both Angurai and Busia. A similar pattern was found for Lambwe Valley although the difference did not reach significance (Tables 2 and 3, Figs. 4, 5, 6, 7). Insectivores were represented in all the land uses and were more abundant in the natural land use than expected by chance or a uniform distribution between the two land uses. Similarly, the granivore guild in Busia and Angurai was more abundant in the natural land use than expected. So, for example, the insectivore comprised 87.5%, 74.2% and 31.0% more species in the natural than the disturbed land use in Angurai, Busia and Lambwe Valley, respectively. The granivore feeding guild comprised 73.9% and 63.6% more species in the natural than the disturbed land use in Angurai and Busia, respectively (Tables 1 and S1, Fig. 7).

**Table 2:** Tests of differences in the expected relative species richness between (a) land uses, sites and their interaction and (b) land uses within each site.

(a) Effect		NDF	DDF	F	P value > F
Landuse		1	61	51.608	1.120 × 10^–9^
Site		2	61	2.958	5.942 × 10^–2^
Site × landuse		2	61	8124	7.450 × 10^–4^

(b) Simple effect slices					
Effect	Slice (= site)				
Site × landuse	Angurai	1	61	31.679	4.873 × 10^–7^
Site × landuse	Busia	1	61	24.951	5.229 × 10^–6^
Site × landuse	Lambwe	1	61	1830	1.811 × 10^–1^

NDF and DDF are the numerator and denominator degrees of freedom, respectively.

**Table 3:** Pairwise comparisons of the expected relative species richness between land uses for each site.

Effect	Slice=(site)	Landuse	Landuse	Diff	SE	DF	T	P >|T|	AdjP
Site × landuse	Angurai	Modified	Natural	− 2.230	0.396	61	− 5.628	4.87 × 10^–7^	0
Site × landuse	Busia	Modified	Natural	− 1.435	0.287	61	− 4.995	5.23 × 10^–6^	0
Site × landuse	Lambwe	Modified	Natural	− 0.375	0.277	61	− 1.353	1.811 × 10^–1^	0.188

*Diff* estimated difference, *Adjp* adjusted P-value.

Species richness in each of the nine guilds found on the disturbed land use was relatively low, thus, only one species represented the frugivore (*Pycnonotus barbatus*) and the insectivore /nectivore *Cinnyris chloropygius* guilds in Angurai. Similarly, in Busia, only *Elanus axillaris* represented the carnivore guild whereas *Francolinus sephaena* represented the omnivore guild in the disturbed land uses. Similarly, in Lambwe Valley, *Lanius excubitoroides* represented the insectivore/carnivore and *Cinnyris pulchella* the nectrivore guild in the disturbed land uses. Except the insectivore in Lambwe Valley, and the granivore in Busia, all the other guilds in the disturbed land use comprised less than four species each (Tables 1 and S1, Fig. 7).

Although the natural and disturbed landscapes supported some guilds that were unique to them, the insectivore/wax feeders were uncommon across the study sites with only one species (Greater honeyguide *Indicator indicator*) being restricted to the natural landscapes in both Angurai and Busia. Two species belonging to the carnivore guild (Black goshawk *Accipiter melanoleucus* and Greyish eagle-owl *Bubo cinerascens*) were recorded in the natural landscape in Angurai. In contrast, in Lambwe Valley, only one raptor species (Eurasian hobby *Falco subbuteo*) was recorded in the natural landscape whereas four raptor species (Black-chested snake-eagle *Circaetus pectoralis*, Black-shouldered kite *Elanus axillaris*, Gabar goshawk *Micronisus gabar* and Long-crested eagle *Lophaetus occipitalis*) were recorded in the disturbed landscape. The number of species in this guild varied significantly between the natural and the disturbed landscapes but none of its members was observed in Busia (Tables 1 and S1, Fig. 7).

The frugivore guild was the least affected by land use disturbance in all the sites, while granivores and insectivores were the most affected with significantly greater diversity in the natural than the disturbed landscapes at all the three sites (Tables 1 and S2, Fig. 7). The species richness of omnivores varied significantly between the natural and disturbed landscapes in both Angurai and Busia but not in Lambwe Valley. Unlike in Busia, the insectivore/nectarivore guild varied significantly between land use types in Angurai. This guild was completely absent in the natural landscape in Lambwe Valley and only one of its species (purple-banded sunbird *Nectarinia bifasciatus*) was recorded in the disturbed landscape there (Tables 1 and S2, Fig. 7).

Unlike in the disturbed sites, guilds in the natural areas of Angurai and Busia were comparable, sharing 26.5% of their species. Although these two sites were predominantly agricultural, their disturbed landscapes had dissimilar species composition for each guild, with the Ss score for the two sites ranging from 0 to 0.24, perhaps reflecting spatial variability in the impacts of land use on their habitats and vegetation composition that support the avifauna (Tables 1 and S2, Fig. 7).

The species richness and Sorensen’s Similarity Index for each feeding guild in the natural and disturbed land use in Angurai, Busia and Lambwe Valley is summarized in Table 1 and in Figs. 6 and 7. The Similarity Index between guilds in the natural and disturbed land uses was low, and ranged from 0.15 to 0.44 for insectivores and frugivores in Busia, and from 0.18 to 0.57 for omnivores and insectivores/nectrivores in Angurai. By contrast, all the guilds in Lambwe Valley exhibited complete dissimilarity between the natural and disturbed land uses, corresponding to a Sorensen’s Similarity Index of zero. The carnivore and the insectivore/wax feeder guilds had the least similarity index between the natural and disturbed land uses for all the three sites, whereas the frugivore guild in Busia and the insectivore/nectrivore guild in Angurai had the highest similarity indices.

The expected species richness differed between land uses and the pattern of the difference varied between sites. Species richness was significantly higher in the natural than the disturbed landscape in Busia and Angurai but was similar between both landscapes in Lambwe Valley (Tables 2 and 3, Fig. 7).

The proportions of both the insectivore and granivore guilds in the natural and disturbed land uses were significantly greater than expected in all the sites. Compared to Angurai and Busia, the insectivore guild was proportionately more abundant than expected in the disturbed land use in Lambwe Valley. Similarly, the proportion of the insectivore guild in the natural land use was more than expected for all the sites. However, the proportion for the granivore guild was less than expected for Lambwe Valley, but more than expected for both Angurai and Busia (Tables 2 and 3, Figs. 6 and 7).

### Influences of range size, foraging style and threat level or trend on species use of disturbed and natural areas

**Figure 8:**
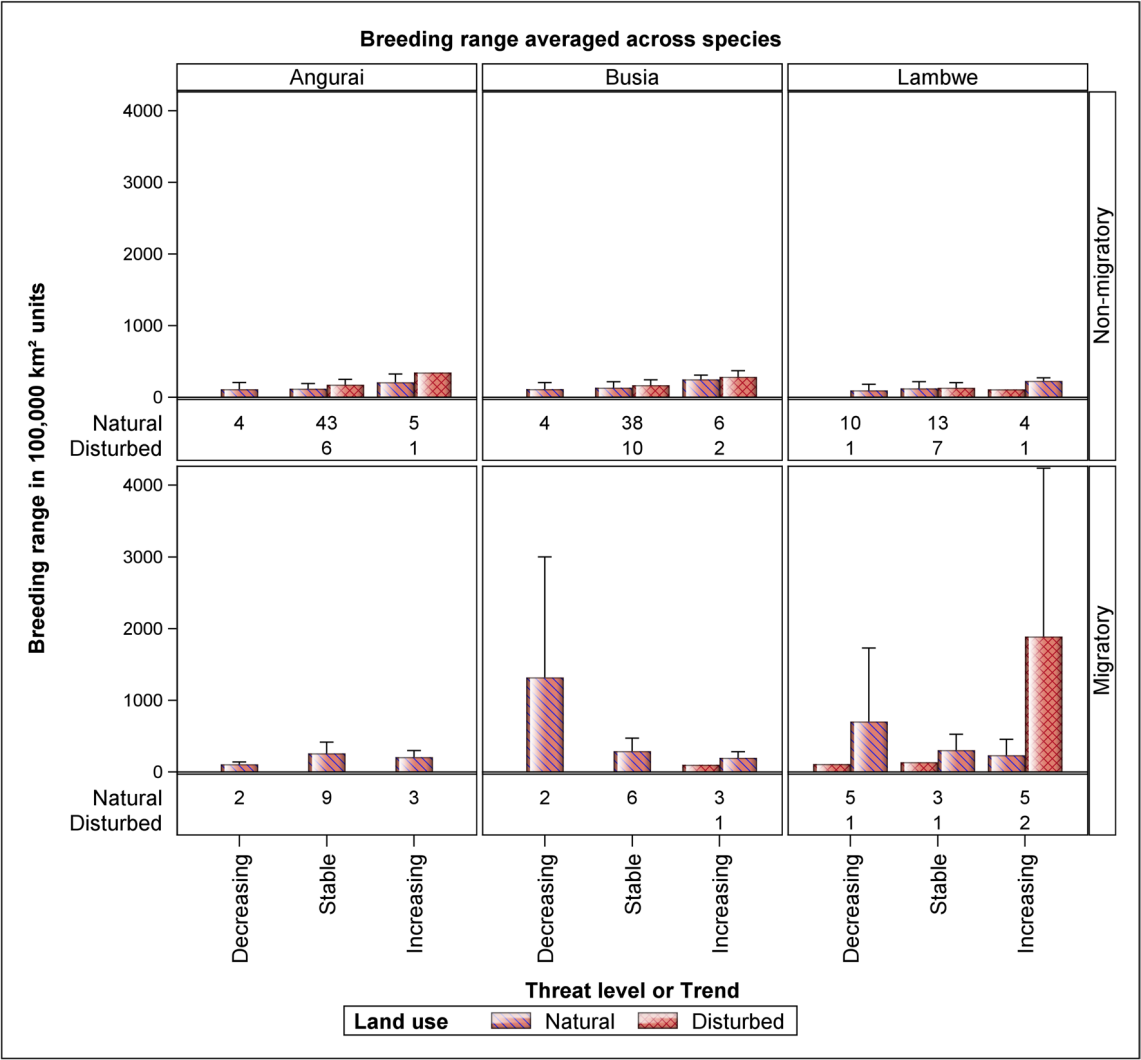
The average range size and its standard deviation calculated across all the species and the total number of all the species found in the disturbed and natural land uses, separately for each unique combination of study site, foraging style (non-migratory, migratory–includes nomadism, partial or altitudinal migration) and threat level or trend (decreasing, stable or increasing).

Migratory species generally have far larger range sizes than non-migrants, consistent with expectations. Most species, irrespective of their breeding range sizes or foraging styles, were present in the natural land use across all the three sites. However, there were notable differences in their presence in the disturbed land use, particularly related to threat level or trend. For species with decreasing trends, only a single migrant and one non-migrant were found in the disturbed land use in Lambwe Valley, and none in Busia or Angurai. For species with stable trends, a greater presence was noted in disturbed areas: Lambwe Valley had 7 non-migrant species, Busia had 10, and Angurai had 6. In contrast, only one migrant species with a stable trend was recorded in the disturbed land use in Lambwe Valley. For species with increasing trends in disturbed land use, the numbers were lower: Lambwe Valley recorded 1 non-migrant species, Busia 2, and Angurai 1. Among the migrants with increasing trends, 2 species were present in the disturbed land use in Lambwe, 1 in Busia, and none in Angurai (Fig. 8). These patterns highlight the nuanced relationship between species' migratory status and threat levels, and their use of disturbed versus natural habitats and how such use is altered by the degree of habitat disturbance or proximity of disturbed habitats to natural habitats.

## Discussion

The main message of this study is that converting even degraded habitats to intensive human use, will markedly reduce avian species richness. This indicates that land use change can have, and usually has, significant adverse consequences for bird species richness and diversity. Our findings corroborate those of earlier studies^9,50,51^ that species composition may change in such a way that specialized species adapted to pristine habitats may disappear, as evidenced by the absence of large ground-foraging birds in the disturbed landscape. In particular, species less able to exploit disturbed landscapes, or cope with human presence, can be expected to disappear faster as human settlement and cultivation expand in disturbed landscapes. Accordingly, preservation of pristine and restoration of degraded habitats and sustaining habitat heterogeneity and connectivity should be central goals of land use planning and management to minimize impacts of intensifying human disturbances on species richness and diversity. Monitoring changes in species richness and diversity, especially of the insectivore and granivore guilds, can provide useful indications of the influences of changing land use on bird species.

The distinct patterns we observed in bird species’ habitat use within the disturbed land use near the Ruma National Park in Lambwe Valley provide valuable insights into the relationship between birds and protected areas. The presence of only one decreasing migrant and non-migrant species in this relatively less disturbed land use, and their absence in the more disturbed Busia and Angurai sites, implies a partial dependence on the resources of the protected park and reflects the relatively lower disturbance of the disturbed habitat in Lambwe. This raises the possibility that these species might have been exterminated in Busia and Angurai, highlighting the critical role of protected areas in species conservation. Furthermore, we noted a marked contrast in the behaviour of the non-migratory species compared to the migratory ones, particularly those with stable or increasing trends. Non-migratory species were found in significant numbers in disturbed areas, indicating a certain level of adaptability or resilience to human-altered landscapes, greater fidelity to particular localities or greater constraints in escaping from the altered habitats. In contrast, migratory species with similar trends showed a clear preference for natural land use, effectively avoiding disturbed areas. This pattern underscores the nuanced ways in which species with contrasting life history traits and strategies respond to habitat changes, and the importance of considering these differences in conservation strategies. Notably, the patterns highlight the greater risks non-migratory species face as human use increasingly degrades their habitats.

Species richness was markedly lower in degraded habitats where human activity was the dominant land use than in natural habitats. Degraded areas still maintained high richness, as did natural areas, but species composition in the natural areas was very different to anywhere else. Our results provide nuanced insights illustrating that disturbed habitat patches are significantly influenced by, and depend upon, their immediate environments without which they cannot adequately function as refuges. Indeed, even though a few species will take advantage of the disturbed habitat patches, the resulting diversity is nevertheless likely to be still much lower than that in the original habitats. Moreover, the small disturbed patches are incapable of sustaining their full complement of the new avian biodiversity if all their surrounding habitats are cultivated or otherwise become greatly modified. More specifically, the relatively high species richness in degraded areas may be explained, at least in part, as follows. The newly opened areas, that have lost their former avian inhabitants, offer new habitats for various species that are better adapted to the changed circumstances. For example, in Angurai, a diverse array of commensal birds had made the disturbed habitats their home. The disturbed fields and their scrubby edges in Angurai had abundant grasses used for thatching and cattle-feed along with many weeds that yield a plentiful supply of small seeds, and so attracted a diverse array of estrildid finches. More generally, granivores were quick to take advantage of the abundance of weed seeds after an area had been cleared.

Busia district, another exemplar of extreme land cover change, provides further insights into the reasons underlying the relatively high species richness found in the disturbed areas. There, virtually all of the immediate vicinities of the study plots had been cultivated except for a huge swamp adjacent to Plot 7. This swampland had a notably rich birdlife and was virtually undisturbed, so its avifauna had probably not changed much for centuries. As a result, there were incursions into Plot 7, with adventists regularly visiting the few trees and weedy patches there for foraging. This greatly distorts the apparent importance of the vegetation on Plot 7, which was essentially an extension of the habitat leading from the swamp. Therefore, merely examining the list of species recorded on Plot 7 without considering the influence of the nearby largely intact swampland, will give a grossly misleading impression of its importance. Similarly, Plot 5, a small patch of scrubland, was by itself too small to support a viable bird population even though a few species took refuge there. The neighbouring patch of woodland with more extensive cover, and perhaps food resources, contained a few woodland birds that almost certainly regularly visited the small patch of scrub in Plot 5 whilst foraging.

Notably, by contrast, the natural habitat in Lambwe had many species not found anywhere else. This suggests that if cultivation continues to spread across its nearly 2 million km^2^ of uncultivated arable land, Sub-Saharan Africa can be expected to experience radical reduction in its avian richness. It follows that to maintain high levels of avian diversity in Sub-Saharan Africa conservation efforts should: (1) protect natural habitats of sufficient size for African birds; (2) maintain habitat connectivity by promoting land uses that enhance connectivity; and (3) urgently implement appropriate interventions, when it is still possible to do so, to counteract the ongoing dramatic increase in land use changes associated with the exponential human population increase, escalating consumption and activities in Africa. Further studies should verify whether the connectivity rules developed for other regions apply to this region or suggest how they should be adaptively modified. These studies should also establish (1) the forest species at greater risk of becoming increasingly threatened, (2) species that might be at risk of transitioning from common to threatened if agricultural intensification continues, (3) patch sizes required to protect particular bird communities and (4) what else specifically will be needed to maximise conservation of species diversity in particular localities and to avoid increasing numbers of species becoming globally threatened.

Although we found higher bird species richness in natural than in disturbed savanna sites, other studies have reported disturbed sites with higher species richness for certain guilds than in adjacent undisturbed forest areas^52^. Thus, even though our findings reaffirm and reinforce those of other studies in Sub-Saharan African savannas, our study covered relatively small areas and different habitats may display different patterns of species richness with disturbance gradients due, for example, to specialists and generalist coexisting in less disturbed areas.

Disturbance evidently reduced species richness and diversity based on multiple lines of evidence and consistent with findings of earlier studies^27,53,54^. First, bird species richness and diversity were clearly higher in the natural than the disturbed land use regardless of site. Second, although similar across all the sites, species diversity (H′) was higher for the natural than the disturbed land use for both Angurai (2.1 times) and Busia (1.6 times). Third, species diversity for the natural land use was not only higher than that for the corresponding disturbed land use but was also similar to the overall site diversity because the natural land use contributed most of the diversity for all the sites. Thus, Angurai which had the highest diversity index (H′) for the natural land use also had the lowest diversity for the disturbed land use. Lastly, disturbance also tended to reduce guild diversity. In consequence, the occurrence of the greatest variation in the bird diversity index (H′) between land uses in Angurai, implies a significantly greater effect of land use on bird species diversity there than in the other two sites. Even though only the insectivore and granivore guilds contributed most to species diversity in all the three sites, both guilds had far more species in the natural than the disturbed land use.

Habitat deterioration and loss due to land use change can be expected to have a much stronger adverse impact on bird species with high habitat specificity or occupying disturbed habitats distant from suitable ones^55–57^. Accordingly, the disturbed landscape in Lambwe Valley differed from those in Angurai and Busia in that it consisted of remnant natural vegetation similar to that of the neighbouring Ruma NP, resulting in similar diversity between the disturbed and the natural landscape but with notably more species in the natural habitat. But although the natural habitats had higher diversity than the disturbed habitats, the disturbed landscapes also had a few bird species that were not found in the natural habitats. For example, the disturbed and isolated landscape in Angurai was home to two bird species that were not observed anywhere else. The first species, an insectivore, the striped kingfisher *Halcyon chelicuti*, was likely attracted to its preferred food items associated with the mosaic of locally cultivated crops and patches of remnant natural vegetation. The second species, a granivore, the Brimstone Canary *Serinus sulphuratus*, also has life-history traits and foraging strategies that suit it to life in disturbed habitats. Similarly, to Angurai, only one bird species, the brown-headed Parrot *Poicephalus cryptoxanthus*, a frugivore but often an opportunistic generalist, occurred only in the disturbed landscape in Busia. These birds are naturally gregarious and usually occur in small flocks of about 12 to 50 individuals and are sometimes crop pests but are shy and wary^58^.

Bird species diversity and land use patterns are related through complex feedbacks^22,55,59^. The differences in bird community structure between disturbed and intact landscapes may be attributable primarily to high habitat specificity^27,60^. Guilds with high habitat specificity were generally fewer in disturbed landscapes than species with more generic habitat requirements, which were more widespread, possibly in response to changes in the distribution, availability and quality of different food items and other resources. Responses of birds to habitat changes differ depending on their strategies, with some benefiting from habitat change, while others get threatened by it^61^. Cultivated and bush land in Busia showed high species similarity (Ss = 0.39). This is partly due to close proximity to the township resulting in rapid land cover conversion and intensification of land use in both land uses. Mature tree species in the remnant bush lands are extracted for timber and other commercial uses, increasingly homogenizing the habitats. Consequently, trees are extremely few in the study sites and the major vegetation in the uncultivated areas are shrubby bushes most of which are grazing areas. The most common indigenous tree is *Makhamia sp* that is widespread in the cultivated areas but almost always as sprouting young plants because harvesting does not let them grow to maturity. The *Makhamia* tree is used mainly for constructing houses because it is straight, non-branching and able to bend without breaking, especially the young ones. Most of the indigenous trees were very young, and were represented mostly by seedlings as they were harvested while still very young. Exotic trees were planted along the hedges of a few home compounds.

The Ss index for Lambwe Valley was low because few bird species found in the disturbed landscape were observed in the Ruma NP. The apparently high bird species diversity and low similarity in Lambwe Valley may be due to the persistence of some of the species that originally inhabited the untransformed habitat remnants within the settled and cultivated landscape mosaic plus additional species able to exploit the transformed habitat fragments. Habitat generalists may survive in very small remnant patches because they can use resources from the modified surroundings^15^. This would suggest a subtle array of possible species-specific thresholds along a disturbance gradient, beyond which species with certain vegetation-specific requirements either drop off or are joined by opportunistic species.

In Busia and Angurai, grasslands and thickets in the natural landscapes were preferred by small, insectivorous passerines, whilst insectivorous species appeared able to cope with increased human land-use activities in Lambwe Valley. However, large non-passerine bird species were virtually absent in all the settled areas, despite being present in the national park. These findings are in accord with the prediction^30^ that large ground-nesting species should become increasingly confined to protected areas as land use change progresses at their edges based on empirical evidence of a dramatic loss of bird species in agricultural areas compared to less disturbed savanna inside the Serengeti National Reserve in northern Tanzania. The findings also suggest that species diversity will increase with edge, but some specialists in more natural habitats will be lost. In contrast to Serengeti, where savanna and cultivated habitats were compared, the physical structure of the disturbed grassland and thicket habitats remained largely unaltered compared to the natural Ruma NP in the Lambwe Valley^62^. Thus, these results suggest that positive impacts on general diversity may occur at low levels of disturbance, when structural differences in vegetation are still not yet marked, but the impacts can rapidly reach a threshold beyond which diversity declines precipitously. Further, the low similarity indexes for all guilds for the Lambwe Valley indicate that although diversity increased, the species composition also changed, likely leading to changes in functional attributes of the ecosystem. Such changes may go unnoticed if baseline data are unavailable.

In addition to land use change, direct human activities such as hunting game birds or scaring away birds regarded as pests add more pressure on certain bird species. For example, two ground-foraging gamebird species, the helmeted guinea fowl *Numida meleagris* and Red-necked spurfowl *Francolinus afer* were only recorded inside the Ruma NP. Gregarious birds that invade cultivated fields of sorghum and maize (e.g., red-billed quelea *quelea*) are also trapped in large numbers during the harvest season^63^. Although hunting may have a strong impact on targeted bird species, it alone does not fully explain the absence inside the human-dominated landscape of non-game bird species such as Green wood-hoopoe *Phoeniculus purpureus* and African grey hornbill *Tockus nasutus*.

Larger insectivorous birds preferentially feed on larger invertebrate prey items^64^. Thus, as land management intensifies, the size distribution of food items like carabid beetles—a major part of the diet of many ground-foraging insectivores, shifts towards smaller sizes^65^. In Lambwe Valley, remaining grasslands are intensively grazed all year-round^62^. Therefore, reduced abundance of larger insects may explain the absence of large ground-foraging birds in grasslands in the settled landscapes, especially because grassland birds seem to closely track variation in the abundance of their invertebrate food prey items in both space and time^30,66^.

In conclusion, escalating human population, consumption and activities in Sub-Saharan Africa are associated with rapid land conversion with profound adverse consequences for avifaunal diversity, abundance and species composition. Protection of representative natural habitats and habitat connectivity and restoration of degraded ones are necessary to slow down, halt or reverse the ongoing trend of massive biodiversity loss. Meeting these goals is likely to be exceptionally challenging due to the rapidly increasing human population in Sub-Saharan Africa, which places growing pressures on scarce resources. This requires prioritizing contemporary and future development initiatives centered on agricultural intensification and productivity improvements within current farming systems, all while considering environmental health. Rural development will persist irrespective of tsetse control, posing a monumental challenge in minimizing its adverse environmental impact^67^. Consequently, it is crucial to implement conservation interventions proactively to prevent irreparable damage to natural ecosystems caused by these developments.

### Supplementary Information

Supplementary Information 1.

Supplementary Information 2.

Supplementary Information 3.

Supplementary Information 4.

Supplementary Information 5.

## Data Availability

The datasets generated during and/or analysed during the current study are provided in the Supplementary Materials of this paper.
