# Supplementary material for: Bird species richness and diversity responses to land use change in the Lake Victoria Basin, Kenya
Source: Sci Rep. 2024 Jan 19;14:1711. doi: 10.1038/s41598-024-52107-2 (PMC10798997; doi:10.1038/s41598-024-52107-2)
Supplement: Supplementary file 1 — Supplementary Information 1. [file 41598_2024_52107_MOESM1_ESM.docx]

**LIST OF BIRD SPECIES RECORDED IN LAMBWE VALLEY, ANGURAI AND BUSIA**

**List of birds in Lambwe Valley (x means sighted, otherwise species not sighted on the transect located at the indicated site)**

| **Common english name** | **Site 3: Grasslands in settled areas** | **Site 2,4: Grasslands/Thickets in the National Park/settled areas** | **Site 1,5: Grassland/thicket in protected area** |
| --- | --- | --- | --- |
| Abdim's Stork |  |  | X |
| Abyssinian Scimitarbill |  | X |  |
| Afr. Moustached Warbler | X |  |  |
| Afr. Paradise Flycatcher |  |  | X |
| African Grey Hornbill |  |  | X |
| Angola Swallow |  | X |  |
| Babbler spp. | X |  |  |
| Bare-faced Go-away-bird |  |  | X |
| Barn Swallow |  | X |  |
| Beautiful Sunbird | X |  |  |
| Black Cuckoo Shrike | X |  |  |
| Black-and-White Cuckoo |  | X |  |
| Black-bellied Bustard |  | X |  |
| Black-chested Snake Eagle |  | X |  |
| Black-headed Gonolek | X |  |  |
| Black-lored Babbler |  |  | X |
| Black-necked Weaver |  | X |  |
| Black-shouldered Kite |  | X |  |
| Black-throated Wattle-eye |  |  | X |
| Blue-naped Mousebird |  | X |  |
| Broad-tailed Warbler |  |  | X |
| Brown Parrot |  |  | X |
| Cattle Egret | X |  |  |
| Crowned Lapwing | X |  |  |
| Emerald-sp. Wood-Dove |  | X |  |
| Eurasian Hobby |  |  | X |
| European Bee-eater |  | X |  |
| Fork-tailed Drongo |  | X |  |
| Gabar Goshawk |  | X |  |
| Green Wood-Hoopoe |  |  | X |
| Grey-backed Camaroptera |  | X |  |
| Grey-backed Fiscal | X |  |  |
| Hadada Ibis |  |  | X |
| Helmeted Guineafowl |  |  | X |
| Laughing Dove |  | X |  |
| Lesser Masked Weaver | X |  |  |
| Lesser Striped Swallow | X |  |  |
| Little Swift |  |  | X |
| Long-crested Eagle |  | X |  |
| Northern Black Flycatcher |  | X |  |
| Pale Flycatcher | X |  |  |
| Purple-banded Sunbird |  | X |  |
| Rattling Cisticola | X |  |  |
| Red-billed Quelea | X |  |  |
| Red-eyed Dove |  | X |  |
| Red-faced Crombec |  | X |  |
| Red-fronted Tinkerbird |  |  | X |
| Red-necked Spurfowl |  |  | X |
| Ring-necked Dove |  | X |  |
| Rüppell's Long-t. Starling |  | X |  |
| Scarlet-breasted Sunbird |  | X |  |
| Slate-coloured Boubou |  | X |  |
| Speckled Mousebird |  | X |  |
| Spectacled Weaver |  | X |  |
| Spotted Flycatcher |  |  | X |
| Sulphur-br. Bush-Shrike |  | X |  |
| Superb Starling |  | X |  |
| Tawny-flanked Prinia |  | X |  |
| Wattled Starling |  | X |  |
| White-brow. Robin-Chat |  | X |  |
| White-brow. Scrub-Robin |  | X |  |
| White-headed Saw-wing |  | X |  |
| White-throated Bee-eater |  |  | X |
| Yellow Wagtail |  | X |  |
| Yellow-fronted Canary | X |  |  |
| Yellow-rumped Seed-eater |  |  | X |
| Yellow-spotted Petronia |  |  | X |
| Yellow-throated Longclaw |  | X |  |

**List of Birds found in Angurai** **(x means species sighted, otherwise species not sighted on the plot)**

| **Common name** | **FLGZ, MZCA** | **WDGR, WDBS, WDBS1** |
| --- | --- | --- |
| African black swift |  | X |
| African citril |  | X |
| African firefinch |  | X |
| african moustached warbler |  | X |
| baglafecht weaver |  | X |
| black & white mannikin |  | X |
| black bellied firefinch |  | X |
| black billed barbet |  | X |
| black faced waxbill |  | X |
| black headed batis |  | X |
| black headed gonolek |  | X |
| black headed heron |  | X |
| black headed weaver |  | X |
| black rumped waxbill |  | X |
| black shouldered kite |  | X |
| blue flycatcher |  | X |
| blue spotted wood dove |  | X |
| Brimstone canary | X |  |
| bronze mannikin | X | X |
| bronze sunbird |  | X |
| brow crowned tchagra |  | X |
| brown backed scrub robin |  | X |
| brown throated wattle eye |  | X |
| cabanis greenbul |  | X |
| common bulbul | X | X |
| common waxbill |  | X |
| compact weaver |  | X |
| copper sunbird |  | X |
| crested francolin |  | X |
| croaking cisticola |  | X |
| dark capped yellow warbler |  | X |
| diederik cuckoo |  | X |
| fawn bellied waxbill |  | X |
| great sparrowhawk |  | X |
| greater honeyguide |  | X |
| green headed sunbird |  | X |
| grey backed camaroptera |  | X |
| grey capped warbler |  | X |
| grey headed sparrow |  | X |
| grey winged robin chat |  | X |
| greyish eagle owl |  | X |
| klaas' cuckoo |  | X |
| laughing dove |  | X |
| little greenbul |  | X |
| marsh tchagra |  | X |
| olive bellied sunbird | X | X |
| olive sunbird |  | X |
| paradise flycatcher |  | X |
| parasitic weaver |  | X |
| pigmy kingfisher |  | X |
| purple banded sunbird |  | X |
| purple grenadier |  | X |
| pygmy kingfisher |  | X |
| red billed firefinch | X | X |
| red cheeked cordon bleu |  | X |
| red crested cuckoo |  | X |
| red eyed dove |  | X |
| red faced cisticola |  | X |
| red headed lovebird |  | X |
| red winged warbler |  | X |
| ross's turaco |  | X |
| scarlet chested sunbird |  | X |
| siffling cisticola |  | X |
| singing cisticola | X | X |
| snowy headed robin chat |  | X |
| speckled mousebird |  | X |
| speckled pigeon |  | X |
| spectacled weaver |  | X |
| striped kingfisher | X |  |
| sulphur breasted bush shrike |  | X |
| tambourine dove |  | X |
| tawny flanked prinia | X | X |
| violet backed starling |  | X |
| whistling cisticola |  | X |
| white browed coucal |  | X |
| white browed robin chat |  | X |
| white chinned prinia | X | X |
| white headed saw wing |  | X |
| yellow fronted canary | X | X |
| yellow fronted tinkerbird | X | X |
| yellow rumped tinkerbird | X | X |
| yellow white eye | X | X |
| yellowbill |  | X |

**List of birds found in Busia (x means sighted, otherwise species not sighted on the plot)**

| **Common names** | **PLOTS 1, 2, 4, 3, 6: MODIFIED AREA (CULTIVATED, FALLOW & SETTLED)** | **PLOT S 5, 7: UNMODIFIED AREA (BUSHLAND, SCRUBLAND AND WOODLAND)** |
| --- | --- | --- |
| African black swift |  | X |
| African citril |  | X |
| African moustached warbler |  | X |
| African palm swift |  | X |
| African pied wagtail | X | X |
| Baglafecht weaver | X | X |
| Bar breasted firefinch |  | X |
| Barn swallow |  | X |
| Black & white mannikin |  | X |
| Black headed gonolek |  | X |
| Black headed weaver |  | X |
| Black shouldered kite | X | X |
| Blue headed coucal |  | X |
| Blue spotted wood dove |  | X |
| Brimstone canary |  | X |
| Bronze mannikin | X | X |
| Brown babber |  | X |
| Brown crowned tchagra |  | X |
| Brown parrot | X |  |
| Common bulbul | X | X |
| Common fiscal | X | X |
| Common waxbill |  | X |
| Compact weaver | X | X |
| Copper sunbird | X | X |
| Crested francolin | X | X |
| Dark capped yellow warbler |  | X |
| Diederik cuckoo |  | X |
| Eastern grey plantain eater |  | X |
| Fawn bellied waxbill |  | XX |
| Fawn breasted waxbill |  | X |
| Greater swamp warbler |  | X |
| Grey capped camaroptera |  | X |
| Grey capped warbler |  | X |
| Grey headed sparrow |  | X |
| Grey woodpecker |  | X |
| Hadada ibis |  | X |
| Holub's golden weaver |  | X |
| Klaas' cuckoo |  | X |
| Laughing dove |  | X |
| Lesser honeyguide |  | X |
| Lesser striped swallow |  | X |
| Little bee eater |  | X |
| Marsh tchagra |  | X |
| Olive bellied sunbird |  | X |
| Papyrus canary |  | X |
| Pied crow |  | X |
| Pigmy kingfisher |  | X |
| Pin tailed whydah | X | X |
| Red billed firefinch |  | X |
| Red billed oxpecker |  | X |
| Red cheeked cordon bleu |  | X |
| Red chested sunbird |  | X |
| Red eyed dove |  | X |
| Red faced cisticola | X | X |
| Ruppell's starling |  | X |
| Scarlet chested sunbird | X | X |
| Senegal coucal |  | X |
| Slender billed weaver |  | X |
| Speckled mousebird |  | X |
| Spectacled weaver |  | X |
| Striped kingfisher |  | X |
| Tawny flanked prinia | X | X |
| Triped kingfisher |  | X |
| Tropical boubou | X | X |
| Variable sunbird |  | X |
| White browed coucal |  | X |
| White browed robin chat | X |  |
| White browed scrub robin |  | X |
| Winding cisticola |  | X |
| Woodland kingfisher | X | X |
| Yellow fronted canary | X | X |
| Yellow fronted tinkerbird |  | X |
| Yellow mantled widowbird |  | X |
| Yellow throated leaflove | X |  |
| Yellow throated longclaw | X | X |
| Yellow white eye |  | X |
