# Supplementary material for: Bird species richness and diversity responses to land use change in the Lake Victoria Basin, Kenya
Source: Sci Rep. 2024 Jan 19;14:1711. doi: 10.1038/s41598-024-52107-2 (PMC10798997; doi:10.1038/s41598-024-52107-2)
Supplement: Supplementary file 5 — Supplementary Information 5. [file 41598_2024_52107_MOESM5_ESM.docx]

**Supplementary Information**

**Bird species richness and diversity responses to land use change in the Lake Victoria Basin, Kenya**

S. Mugatha, Joseph O. Ogutu, Hans-Peter Piepho, Joseph M. Maitima.

Authors: S. Mugatha^1^, Joseph O. Ogutu^1,3^, Hans-Peter Piepho^3^, Joseph M. Maitima^1,2^.

^1^International Livestock Research Institute (ILRI), P.O. Box 30709 - 00100, Nairobi, Kenya

^2^Ecodym Africa, P.O. Box 50901 - 00200, Nairobi - Kenya

^3^University of Hohenheim, Institute of Crop Science, Biostatistics Unit, Fruwirthstrasse 23, 70599 Stuttgart, Germany. Tel: +49 -711- 45923022; Fax: +49-711-4459 24345

Corresponding author: Joseph O. Ogutu. Email: [jogutu2007@gmail.com](mailto:s.mugatha@cgiar.org)

**Table S1**. The GPS locations and descriptions of the plots used in the Angurai and Busia study sites. In Angurai disturbed plots are CULT, FLGZ, MZCA and WDGR whereas natural plots are WDBS and WDBS1. In Busia disturbed (cultivated, fallow or settled) plots are PLOTs 1, 2, 3, 4 and 6 whereas natural plots are PLOTS 5 and 7.

| **Site** | **Plot Label** | **UTM Zone** | **Eastings** | **Northings** | **Plot Description** |
| --- | --- | --- | --- | --- | --- |
| Angurai | CULT | 36N | 0647709 | 0080225 | Cultivated plot adjacent to WDBS1, open area without natural cover but with *Lantana camara* thickets nearby. |
|  | FA | 36N | 0623538 | 0050248 |  |
|  | FLGZ | 36N | 0647727 | 0079662 | Flat, open short grassland with no tree cover and *Lantana camara* thickets on the periphery. Partly turned over to sweet potato cultivation, situated in an area of extensive cultivation. |
|  | MZ | 36N | 0647189 | 0079064 |  |
|  | MZCA | 36N | 06476331 | 0079631 | Small, flat area with cassava and recently harvested maize. No natural bush or tree cover, situated in an extensively cultivated area. |
|  | WDBS | 36N | 0647224 | 0078962 | Small area of natural vegetation amid changed forest growth, located among rocky boulders difficult to farm. Some trees reach five to six meters, though much of the original timber has been removed. Unlike WDGR, legumes are well-represented, particularly *Albizia*. These leguminous trees traditionally offer rich nourishment for birds. The undergrowth is dense and rank, retaining humidity with a much gentler gradient, featuring flat areas and a stream.  The soil retains moisture in the accumulated humus and clay substrate. The more open side of the boulder hill is heavily cultivated. The location is heavily cultivated, but grassy slopes and dense thickets downhill from the plot, as well as a naturally vegetated hill adjacent, provide natural cover. This region has the most natural cover among those visited.  The plot is contiguous with a scattering of native trees and dense scrub patches interspersed with native grassland meadows, offering considerable habitat for birds. The understorey is open with a very dense cover of shrubs, many of them armed, providing good shelter for birds. |
|  | WDBS1 | 36N | 0647615 | 0080215 | A small plot of natural secondary dry scrub on a steep, boulder-strewn hill in a heavily cultivated area. Rank growth is present along the bordering stream, forming only a ribbon of natural vegetation with a few trees. |
|  | WDGR | 36N | 0645755 | 0078905 | Broad-leaved scrub, barely four meters high, lacking trees except a few along boundaries. There is no shrubby understorey, only rank grass, indicating a predominantly dry biome. The terrain is well-drained and situated on a steep slope with a constant incline. The vegetation seems limited to a few dominant species, with legumes poorly represented. Evidently, it is secondary growth, as all trees over four meters have already been harvested. The natural vegetation forms a very small area within extensive cultivation, with thicker growth along nearby creeks and small valleys. |
| Busia |  |  |  |  |  |
|  | PLOT 1 | 36N | 0623162 | 0048268 | Contiguous with Plots 2 & 4, Plot 1 is cleared and part of the same general area. Plots 1, 2 & 4 have recently been ploughed up in preparation for planting. |
|  | PLOT 2 | 36N | 0623088 | 0049147 | Contiguous with Plots 1 & 4 and cleared |
|  | PLOT 3 | 36N | 0623082 | 0048669 | An area of fallow with isolated scrubby bushes and a rank hedgerow composed of the exotic *Lantana camara* and *Tithonia*. The plot serves as a feeding ground for gramnivores and a few insectivores, offering limited nesting habitat. |
|  | PLOT 4, | 36N | 0623082 | 0048268 | Contiguous with Plots 1 & 2 and cleared. Plots 1, 2 & 4 were in the same general area and had been recently ploughed up for planting. |
|  | PLOT 5 | 36N | 0623607 | 0047803 | Comprising a small area of low scrub, the site has a few neighbouring tall trees on an adjacent school ground. However, the scrub itself exhibits very low biodiversity, as it is surrounded by completely cleared land. |
|  | PLOT 6 | 36N | 0622881 | 0047811 | A small area of fallow ground, but unlike Plot 3, had no scrub cover. |
|  | PLOT 7 | 36N | 0622861 | 0047139 | The plot includes a few trees, including one *Ficus*, and open scrubby grassland. The chain of vegetation leading from a pristine area of swampland allows many adventists to visit this plot, potentially giving a false impression of its importance for birds. |
|  | SRQP | 36N | 0623624 | 0047782 |  |

### **Table S2**. Species of birds observed in the modified and natural/semi natural habitats in Lambwe Valley, Busia and Angurai study sites in western Kenya during 1998-2004.

| Species | |  |  |  | Sites | | | | | | |
| --- | --- | --- | --- | --- | --- | --- | --- | --- | --- | --- | --- |
|  |  |  | Range in km^2^ (NB=Non Breeding)  (B= Breeding) | Status (Trend) | Foraging Style (M=  Migrant, NM=Not a Migrant) | Lambwe Valley | | Busia | | Angurai |  |
| Common name | Scientific name | Guild |  |  |  | Disturbed | Natural | Disturbed | Natural | Disturbed | Natural |
| Abdim's Stork | *Ciconia abdimii* | insectivore | 13,700,000NB  8,790,000B | Decreasing | M | 0 | 1 | 0 | 0 | 0 | 0 |
| Abyssinian Scimitarbill | *Rhinopomastus minor* | insectivore | 2,960,000B | Decreasing | NM | 0 | 1 | 0 | 0 | 0 | 0 |
| African black swift | *Cypseloides niger* | insectivore | 13,000,000B | Decreasing | PM (partial migrant) | 0 | 0 | 0 | 1 | 0 | 1 |
| East African citril | *Serinus citrinelloides* | granivore | 1,050,000B | Stable | NM | 0 | 0 | 0 | 1 | 0 | 1 |
| African Grey Hornbill | *Tockus nasutus,* | frugivore | 23,500,000B | Stable | NM | 0 | 1 | 0 | 0 | 0 | 0 |
| African Moustached Warbler | *Acrocephalus melanopogon* | insectivore | 13,600,000B | Stable | M | 1 | 0 | 0 | 1 | 0 | 1 |
| African palm swift | *Cypsiurus parvus* | insectivore | 26,500,000B | Increasing | NM | 0 | 0 | 0 | 1 | 0 | 0 |
| African Paradise Flycatcher | *Bradornis microrhynchus* | insectivore | 3,110,000B | Stable | NM | 0 | 1 | 0 | 0 | 0 | 0 |
| African pied wagtail | *Motacilla alba yarrellii* | insectivore | 25,100,000B | Stable | NM | 0 | 0 | 1 | 1 | 0 | 0 |
| Angola Swallow | *Hirundo angolensis* | insectivore | 4,940,000B | Increasing | M | 0 | 1 | 0 | 0 | 0 | 0 |
| Baglafecht Weaver | *Ploceus baglafecht* | insectivore | 6,710,000B | Stable | NM | 0 | 0 | 1 | 1 | 0 | 1 |
| Bar breasted firefinch | *Lagonosticta rufopicta* | granivore | 8,150,000B | Stable | NM | 0 | 0 | 0 | 1 | 0 | 0 |
| Bare faced Go-away-bird | *Corythaixoides personatus* | frugivore | 224,000B | Stable | NM | 0 | 1 | 0 | 0 | 0 | 0 |
| Barn swallow | *Hirundo rustica* | insectivore | 251,000,000B | Decreasing | M | 0 | 1 | 0 | 1 | 0 | 0 |
| Beautiful Sunbird | *Cinnyris pulchella* | nectarivore | 10,300,000B | Stable | NM | 1 | 0 | 0 | 0 | 0 | 0 |
| Black & white mannikin | *Spermestes bicolor* | granivore | 4,200,000B | Stable | NM | 0 | 0 | 0 | 1 | 0 | 1 |
| Black and White Cuckoo | *Cuculus clamosus* | insectivore/ frugivore | 21,800,000NB | Stable | M | 0 | 1 | 0 | 0 | 0 | 0 |
| Black bellied Bustard | *Lissotis melanogaster* | omnivores | 20,900,000B  21,400,000NB | Decreasing | NM | 0 | 1 | 0 | 0 | 0 | 0 |
| Black bellied firefinch | *Lagonosticta rara* | granivore | 4,450,000B | Stable | NM | 0 | 0 | 0 | 0 | 0 | 1 |
| Black billed barbet | *Lybius guifsobalito* | insectivore/ frugivore | 3,750,000B | Increasing | NM | 0 | 0 | 0 | 0 | 0 | 1 |
| Black chested Snake Eagle | *Circaetus pectoralis)* | Carnivore | 13,900,000B  14,300,000NB | Stable | M (nomadic) | 0 | 1 | 0 | 0 | 0 | 0 |
| Black Cuckoo Shrike | *Coracina novaehollandiae* | insectivore | 11,000,000B  16,600,000NB | Decreasing | M | 1 | 0 | 0 | 0 | 0 | 0 |
| Black faced waxbill | *Estrilda atricapilla* | granivore | 2,700,000B | Stable | NM | 0 | 0 | 0 | 0 | 0 | 1 |
| Black headed batis | *Batis minor* | insectivore | 868,000B | Decreasing | NM | 0 | 0 | 0 | 0 | 0 | 1 |
| Black headed gonolek | *Laniarius erythrogaster* | insectivore | 5,910,000B | Stable | NM | 1 | 0 | 0 | 1 | 0 | 1 |
| Black headed heron | *Ardea melanocephala* | insectivore/ carnivore | 26,000,000B | Increasing | M | 0 | 0 | 0 | 0 | 0 | 1 |
| Black headed weaver | *Ploceus melanocephalus* | insectivore/ granivore | 12,300,000B | Stable | NM | 0 | 0 | 0 | 1 | 0 | 1 |
| Black lored Babbler | *Turdoides melanops* | insectivore | 422,000B | Decreasing | NM | 1 | 1 | 0 | 0 | 0 | 0 |
| Black necked Weaver | *Ploceus nigricollis* | insectivore | 7,060,000B | Stable | NM | 0 | 1 | 0 | 0 | 0 | 0 |
| Black rumped waxbill | *Estrilda troglodytes* | granivore | 8,660,000B | Stable | NM | 0 | 0 | 0 | 0 | 0 | 1 |
| Black shouldered kite | *Elanus axillaris* | Carnivore | 10,000,000B | Increasing | M (nomadic) | 0 | 1 | 1 | 1 | 0 | 1 |
| Black throated Wattle-eye | *Platysteira peltata* | insectivore | 7,350,000B | Decreasing | M | 0 | 1 | 0 | 0 | 0 | 0 |
| Blue flycatcher | *Elminia longicauda* | insectivore | 8,760,000B | Stable | NM | 0 | 0 | 0 | 0 | 0 | 1 |
| Blue headed coucal | *Centropus monachus* | omnivores | 9,870,000B | Stable | NM | 0 | 0 | 0 | 1 | 0 | 0 |
| Blue naped Mousebird | *Urocolius macrourus* | frugivore | 14,700,000B | Decreasing | NM | 0 | 1 | 0 | 0 | 0 | 0 |
| Blue spotted wood dove | *Turtur afer* | granivore | 19,100,000B  18,900,000NB | Stable | M | 0 | 0 | 0 | 1 | 0 | 1 |
| brimstone canary | *Crithagra sulpurata* | granivore | 8,700,000B | Stable | NM | 0 | 0 | 0 | 1 | 1 | 0 |
| Broad tailed Warbler | *Catriscus brevirostris* | insectivore | 11,700,000B | Decreasing | NM | 0 | 1 | 0 | 0 | 0 | 0 |
| Bronze mannikin | *Lonchura cucullata* | granivore | 23,600,000B | Stable | NM | 0 | 0 | 1 | 1 | 1 | 1 |
| Bronze sunbird | *Nectarinia kilimensis* | insectivore | 4,160,000B | Stable | NM | 0 | 0 | 0 | 0 | 0 | 1 |
| Brown babbler | *Pellorneum fuscocapillum,* | omnivores | 9,360,000B | Stable | NM | 0 | 0 | 0 | 1 | 0 | 0 |
| Brown backed scrub robin | *Cercotrichas hartlaubi* | insectivore | 3,170,000B | Stable | NM | 0 | 0 | 0 | 0 | 0 | 1 |
| Brown crowned tchagra | *Tchagra australis* | insectivore | 15,100,000B | Stable | NM | 0 | 0 | 0 | 1 | 0 | 1 |
| Brown parrot | *Pocephalus meyeri* | frugivore | 10,400,000B | Stable | NM | 0 | 1 | 1 | 0 | 0 | 0 |
| Brown throated wattle eye | *Platysteira cyanea* | insectivore | 10,700,000B | Stable | NM | 0 | 0 | 0 | 0 | 0 | 1 |
| Cabanis greenbul | *Phyllastrephus cabanisi* | insectivore | 4,130,000B | Stable | NM | 0 | 0 | 0 | 0 | 0 | 1 |
| Cattle Egret | *Bubulcus ibis* | insectivore | 355,000,000B  349,000,000NB | Increasing | M | 1 | 0 | 0 | 0 | 0 | 0 |
| Common bulbul | *Pycnonotus barbatus,* | frugivore | 34,600,000B | Increasing | NM | 0 | 0 | 1 | 1 | 1 | 1 |
| Common fiscal | *Lanius collaris* | insectivore | 22,400,000B | Increasing | NM | 0 | 0 | 1 | 1 | 0 | 0 |
| Common waxbill | *Estrilda astrild* | granivore | 22,200,000B | Stable | NM | 0 | 0 | 0 | 1 | 0 | 1 |
| Compact weaver | *Ploceus superciliosus* | granivore | 9,590,000B | Stable | NM | 0 | 0 | 1 | 1 | 0 | 1 |
| Copper sunbird | *Cinnyris cupreus* | insectivore/ nectarivore | 16,400,000B | Stable | NM | 0 | 0 | 1 | 1 | 0 | 1 |
| Crested francolin | *Ortygonis sephaena* | omnivore | 8,750,000B | Stable | NM | 0 | 0 | 1 | 1 | 0 | 1 |
| Croaking cisticola | *Cisticola natalensis,* | insectivore | 20,400,000B | Stable | NM | 0 | 0 | 0 | 0 | 0 | 1 |
| Crowned Lapwing | *Vanellus coronatus* | insectivore | 11,100,000B | Increasing | NM | 1 | 0 | 0 | 0 | 0 | 0 |
| Dark capped yellow warbler | *Iduna natalensis massaica* | insectivore | 11,100,000B | Stable | NM | 0 | 0 | 0 | 1 | 0 | 1 |
| Diederik cuckoo | *Chrysococcyx caprius* | insectivore | 30,000,000B  5,820,000NB | Stable | M | 0 | 0 | 0 | 1 | 0 | 1 |
| Eastern grey plantain eater | *Crinifer zonurus,* | frugivore | 3,580,000B | Stable | NM | 0 | 0 | 0 | 1 | 0 | 0 |
| Grey-capped Emerald/Emerald Wood dove | *Chalcophaps indica* | granivore | 21,300,00B | Decreasing | NM | 0 | 1 | 0 | 0 | 0 | 0 |
| Eurasian Hobby | *Falco subbuteo* | Carnivore | 52,600,000B  49,300,000NB | Decreasing | M | 0 | 1 | 0 | 0 | 0 | 0 |
| European Bee-eater | *Merops apiaster* | insectivore | 55,700,000B  12,800,000NB | Stable | M | 0 | 1 | 0 | 0 | 0 | 0 |
| fawn breasted waxbill | *Estrilda paludicola* | granivore | 7,530,000B | Stable | NM | 0 | 0 | 0 | 1 | 0 | 0 |
| Fork tailed Drongo | *Dicrurus adsimilis,* | insectivore | 25,900,000B | Stable | NM | 0 | 1 | 0 | 0 | 0 | 0 |
| Gabar Goshawk | *Micronisus gabar* | Carnivore | 26,100,000B | Stable | NM | 0 | 1 | 0 | 0 | 0 | 0 |
| Great/Black sparrowhawk | *Accipiter melanoleucus* | Carnivore | 22,600,000B  23,800,000NB | Decreasing | NM | 0 | 0 | 0 | 0 | 0 | 1 |
| Greater honeyguide | *Indicator indicator* | insectivore/  wax feeder | 24,600,000B | Increasing | NM | 0 | 0 | 0 | 0 | 0 | 1 |
| Greater swamp warbler | *Acrocephalus rufescens* | insectivore | 12,300,000B | Stable | NM | 0 | 0 | 0 | 1 | 0 | 0 |
| Green headed sunbird | *Cyanomitra verticalis* | insectivore/nectarivore | 10,200,000B | Stable | NM | 0 | 0 | 0 | 0 | 0 | 1 |
| Green Wood-Hoopoe | *Phoeniculus purpureus* | insectivore | 22,700,000B | Decreasing | NM | 0 | 1 | 0 | 0 | 0 | 0 |
| Grey backed/Bleating camaroptera | *Camaroptera brachyura* | insectivore | 25,400,000B | Increasing | NM | 0 | 1 | 0 | 0 | 0 | 1 |
| Grey capped camaroptera (Recent studies suggest it is equivalent to the Bleating/Grey-backed camaroptera) | *Camaroptera brevicaudata* | insectivore | 25,400,000B | Increasing | NM | 0 | 0 | 0 | 1 | 0 | 0 |
| Grey capped warbler | *Eminia lepida* | insectivore | 1,000,000B | Stable | NM | 0 | 0 | 0 | 1 | 0 | 1 |
| Grey headed sparrow | *Passer griseus* | granivore | 18,800,000B | Stable | NM | 0 | 0 | 0 | 1 | 0 | 1 |
| Grey winged robin chat/akalat | *Sheppardia polioptera* | insectivore | 7,290,000B | Decreasing | NM | 0 | 0 | 0 | 0 | 0 | 1 |
| Grey woodpecker | *Dendropicos goertae* | insectivore | 12,400,000B | Stable | NM | 0 | 0 | 0 | 1 | 0 | 0 |
| Grey-backed Fiscal | *Lanius excubitoroides* | insectivore/  carnivore | 8,430,000B  5,310,000NB | Stable | NM | 1 | 0 | 0 | 0 | 0 | 0 |
| Greyish eagle owl | *Bubo cinerascens* | Carnivore | 12,600,000B | Stable | NM | 0 | 0 | 0 | 0 | 0 | 1 |
| Hadada ibis | *Bostrychia hagedash* | insectivore | 25,000,000B | Increasing | NM | 0 | 1 | 0 | 1 | 0 | 0 |
| Helmeted Guineafowl | *Numida meleagris* | omnivores | 24,500,000B | Stable | NM | 0 | 1 | 0 | 0 | 0 | 0 |
| Holub'sgolden weaver | *Ploceus xanthops* | insectivore | 7,700,000B | Stable | NM | 0 | 0 | 0 | 1 | 0 | 0 |
| Klaas' cuckoo | *Chrysococcyx klaas* | insectivore | 25,500,000B  14,600,000NB | Stable | M | 0 | 0 | 0 | 1 | 0 | 1 |
| Laughing dove | *Spilopelia senegalensis* | granivore | 64,400,000B | Stable | M | 0 | 0 | 0 | 1 | 0 | 1 |
| Lesser honeyguide | *Indicator minor* | insectivore/  wax feeder | 24,500,000B | Stable | NM | 0 | 0 | 0 | 1 | 0 | 0 |
| Lesser Masked Weaver | *Ploceus intermedius* | granivore | 10,500,000B | Stable | NM | 1 | 0 | 0 | 0 | 0 | 0 |
| Lesser striped swallow | *Cecropis abyssinica* | insectivore | 23,200,000B  21,000,000NB | Increasing | M | 1 | 0 | 0 | 1 | 0 | 0 |
| Little bee eater | *Merops pusillus* | insectivore | 22,000,000B | Decreasing | NM | 0 | 0 | 0 | 1 | 0 | 0 |
| Little greenbul | *Eurillas virens* | frugivore | 9,830,000B | Stable | M (altitudinal migrant) | 0 | 0 | 0 | 0 | 0 | 1 |
| Little Swift | *Apus affinis* | insectivore | 60,600,000B  55,800,000NB | Increasing | M | 0 | 1 | 0 | 0 | 0 | 0 |
| Long-crested Eagle | *Lophaetus occipitalis)* | Carnivore | 24,600,000B | Increasing | NM | 0 | 1 | 0 | 0 | 0 | 0 |
| Marsh tchagra | *Bocagia minuta* | insectivore | 14,400,000B | Decreasing | NM | 0 | 0 | 0 | 1 | 0 | 1 |
| Northern Black Flycatcher | *Melaenornis edolioides,* | insectivore | 9,780,000B | Stable | NM | 0 | 1 | 0 | 0 | 0 | 0 |
| Olive bellied sunbird | *Cinnyris chloropygius* | insectivore/  nectarivore | 9,030,000B | Stable | NM | 0 | 0 | 0 | 1 | 1 | 1 |
| Olive sunbird | *Cinnyris jugularis* | insectivore/  nectarivore | 22,400,000B | Stable | NM | 0 | 0 | 0 | 0 | 0 | 1 |
| Pale Flycatcher | *Agricola pallidus* | insectivore | 20,900,000B | Stable | NM | 1 | 0 | 0 | 0 | 0 | 0 |
| Papyrus canary | *Crithagra koliensis* | frugivore | 261,000B | Decreasing | NM | 0 | 0 | 0 | 1 | 0 | 0 |
| Paradise flycatcher | *Terpsiphone viridis* | insectivore | 26,800,000B  25,000,000NB | Stable | M | 0 | 0 | 0 | 0 | 0 | 1 |
| Parasitic weaver /Cuckoo finch | *Anomalospiza imberbis* | granivore | 17,300,000B | Stable | NM | 0 | 0 | 0 | 0 | 0 | 1 |
| Pied crow | *Corvus albus* | omnivores | 32,900,000B | Stable | NM | 0 | 0 | 0 | 1 | 0 | 0 |
| Pigmy kingfisher | *Ispidina picta* | insectivore | 21,800,000B  15,700,000NB | Stable | M | 0 | 0 | 0 | 1 | 0 | 1 |
| Pin tailed whydah | *Vidua macroura,* | granivore | 26,600,000B | Stable | NM | 0 | 0 | 1 | 1 | 0 | 0 |
| Purple banded Sunbird | *Cinnyris bifasciatus* | insectivore/  nectarivore | 9,470,000B | Stable | NM | 0 | 1 | 0 | 0 | 0 | 1 |
| Purple grenadier | *Granatina ianthinogaster* | granivore | 2,510,000B | Stable | NM | 0 | 0 | 0 | 0 | 0 | 1 |
| Rattling Cisticola | *Cisticola chiniana* | insectivore | 10,800,000B | Stable | NM | 1 | 0 | 0 | 0 | 0 | 0 |
| Red-billed firefinch | *Lagonosticta senegala* | granivore | 25,600,000B | Stable | NM | 0 | 0 | 0 | 1 | 1 | 1 |
| Red-billed oxpecker | *Buphagus erythrorhynchus* | insectivore | 8,930,000B | Decreasing | NM | 0 | 0 | 0 | 1 | 0 | 0 |
| Red-billed Quelea | *Quelea quelea* | granivore | 25,800,000B | Stable | NM | 1 | 0 | 0 | 0 | 0 | 0 |
| Red-cheeked cordon bleu | *Uraeginthus bengalus* | granivore | 15,900,000B | Stable | NM | 0 | 0 | 0 | 1 | 0 | 1 |
| Red-chested sunbird | *Cinnyris erythrocerca* | insectivore/  nectarivore | 1,420,000B | Stable | NM | 0 | 0 | 0 | 1 | 0 | 0 |
| Red-crested cuckoo | *Cuculus solitarius* | granivore | 22,400,000B  10,500,000NB | Stable | M | 0 | 0 | 0 | 0 | 0 | 1 |
| Red-eyed dove | *Streptopelia semitorquata* | granivore | 26,000,000B | Increasing | M | 0 | 1 | 0 | 1 | 0 | 1 |
| Red-faced cisticola | *Cisticola erythrops* | insectivore | 19,400,000B | Stable | NM | 0 | 0 | 1 | 1 | 0 | 1 |
| Red-fronted Tinkerbird | *Pogoniulus pusillus* | insectivore/  frugivore | 6,880,000B | Stable | NM | 0 | 1 | 0 | 0 | 0 | 0 |
| Red-headed lovebird | *Agapornis pullarius.* | granivore | 8,360,000B | Decreasing | M | 0 | 0 | 0 | 0 | 0 | 1 |
| Red-winged warbler/prinia | *Prinia erythroptera* | insectivore | 13,900,000B | Stable | NM | 0 | 0 | 0 | 0 | 0 | 1 |
| Red-faced Crombec | *Sylvietta whytii* | insectivore | 4,170,000B | Decreasing | NM | 0 | 1 | 0 | 0 | 0 | 0 |
| Red-necked Spurfowl/ Francolin | *Pternistis afer* | omnivore | 9,400,000B | Decreasing | NM | 0 | 1 | 0 | 0 | 0 | 0 |
| Ring-necked Dove | *Streptopelia capicola),* | granivore | 15,100,000B | Increasing | M | 0 | 1 | 0 | 0 | 0 | 0 |
| Ross's turaco | *Musophaga rossae* | frugivore | 6,370,000B | Stable | NM | 0 | 0 | 0 | 0 | 0 | 1 |
| Rueppell's long-tailed/glossy Starling | *Lamprotornis purpuropterus* | omnivores | 3,670,000B | Unknown | NM | 0 | 1 | 0 | 1 | 0 | 0 |
| Scarlet-chested Sunbird | *Chalcomitra senegalensis* | insectivore/  nectarivore | 22,300,000B | Stable | NM | 0 | 0 | 1 | 1 | 0 | 1 |
| Scarlet-breasted sunbird/Javan sunbird | *Aethopyga mystacalis* | nectarivore | 177,000B | Decreasing | NM | 0 | 1 | 0 | 0 | 0 | 0 |
| Senegal coucal | *Centropus senegalensis* | omnivores | 23,900,000B | Stable | NM | 0 | 0 | 0 | 1 | 0 | 0 |
| Siffling/Short-winged cisticola | *Cisticola brachypterus* | insectivore | 17,900,000B | Stable | NM | 0 | 0 | 0 | 0 | 0 | 1 |
| Singing cisticola | *Cisticola cantans* | insectivore | 16,000,000B | Stable | NM | 0 | 0 | 0 | 0 | 1 | 1 |
| Slate-coloured Boubou | *Laniarius funebris* | insectivore | 2,390,000B | Stable | NM | 0 | 1 | 0 | 0 | 0 | 0 |
| Slender billed weaver | *Ploceus pelzelni* | insectivore | 6,040,000B | Stable | NM | 0 | 0 | 0 | 1 | 0 | 0 |
| Snowy-headed robin chat | *Cossypha niveicapilla* | insectivore | 10,900,000B | Stable | NM | 0 | 0 | 0 | 0 | 0 | 1 |
| Speckled mousebird | *Colius striatus* | frugivore | 16,300,000B | Increasing | NM | 0 | 1 | 0 | 1 | 0 | 1 |
| Speckled pigeon | *Columba guinea* | granivore | 25,500,000B | Stable | NM | 0 | 0 | 0 | 0 | 0 | 1 |
| Spectacled weaver | *Ploceus ocularis* | insectivore | 12,500,000B | Stable | NM | 0 | 1 | 0 | 1 | 0 | 1 |
| Spotted Flycatcher | *Muscicapa striata,* | insectivore | 32,400,000B  19,500,000NB | Decreasing | M | 0 | 1 | 0 | 0 | 0 | 0 |
| Striped kingfisher | *Halcyon chelicuti* | insectivore | 22,400,000B | Stable | NM | 0 | 0 | 0 | 1 | 1 | 0 |
| Sulphur/Orange breasted bush shrike | *Chlorophoneus sulfureopectus* | insectivore | 20,400,000B | Stable | NM | 0 | 0 | 0 | 0 | 0 | 1 |
| Yellow-bellied waxbill | *Coccopygia quartinia* | insectivore/ granivore | 3,240,000B | Stable | NM | 0 | 0 | 0 | 1 | 0 | 1 |
| Total |  |  |  |  |  | 13 | 41 | 13 | 60 | 7 | 67 |

**S1 Data**. The presence/absence data for all the sighted species summarized at the plot or transect (group) level in the three study sites in western Kenya during 1998-2004.

**S2 Data**. The sightings of the species summarized by site, land use, habitat type and plot or transect in the three study sites in western Kenya during 1998-2004. The description of each land use is also provided.

**S3 Data**. The presence/absence data for all the sighted species summarized by site, land use, and habitat type in the three study sites in western Kenya during 1998-2004.

**S4 Data**. The presence/absence of each species in the disturbed and natural land uses in each of the three study sites in western Kenya during 1998-2004. The guild, breeding range and non-breeding range (when available), threat level or trend and foraging style are also provided for each species.
